# Neuronal Control of Metabolism through Nutrient-Dependent Modulation of Tracheal Branching

**DOI:** 10.1016/j.cell.2013.12.008

**Published:** 2014-01-16

**Authors:** Gerit A. Linneweber, Jake Jacobson, Karl Emanuel Busch, Bruno Hudry, Christo P. Christov, Dirk Dormann, Michaela Yuan, Tomoki Otani, Elisabeth Knust, Mario de Bono, Irene Miguel-Aliaga

**Affiliations:** 1 Gut Signalling and Metabolism Group, MRC Clinical Sciences Centre, Imperial College London, Du Cane Road, London W12 0NN, UK; 2 MRC Laboratory of Molecular Biology, Francis Crick Avenue, Cambridge CB2 0QH, UK; 3 Department of Zoology, University of Cambridge, Downing Street, Cambridge CB2 3EJ, UK; 4 Max Planck Institute of Molecular Cell Biology and Genetics, Pfotenhauerstrasse 108, 01307 Dresden, Germany

## Abstract

During adaptive angiogenesis, a key process in the etiology and treatment of cancer and obesity, the vasculature changes to meet the metabolic needs of its target tissues. Although the cues governing vascular remodeling are not fully understood, target-derived signals are generally believed to underlie this process. Here, we identify an alternative mechanism by characterizing the previously unrecognized nutrient-dependent plasticity of the *Drosophila* tracheal system: a network of oxygen-delivering tubules developmentally akin to mammalian blood vessels. We find that this plasticity, particularly prominent in the intestine, drives—rather than responds to—metabolic change. Mechanistically, it is regulated by distinct populations of nutrient- and oxygen-responsive neurons that, through delivery of both local and systemic insulin- and VIP-like neuropeptides, sculpt the growth of specific tracheal subsets. Thus, we describe a novel mechanism by which nutritional cues modulate neuronal activity to give rise to organ-specific, long-lasting changes in vascular architecture.

## Highlights

• The *Drosophila* tracheal system exhibits nutrient-dependent plasticity
• Tracheal plasticity is organ specific and metabolically significant
• Nutrient- and hypoxia-responsive neurons drive adaptive tracheation
• Distinct insulin-like and Pdf neuropeptides control organ-specific tracheal branching

## Graphical Abstract

**Figure:**
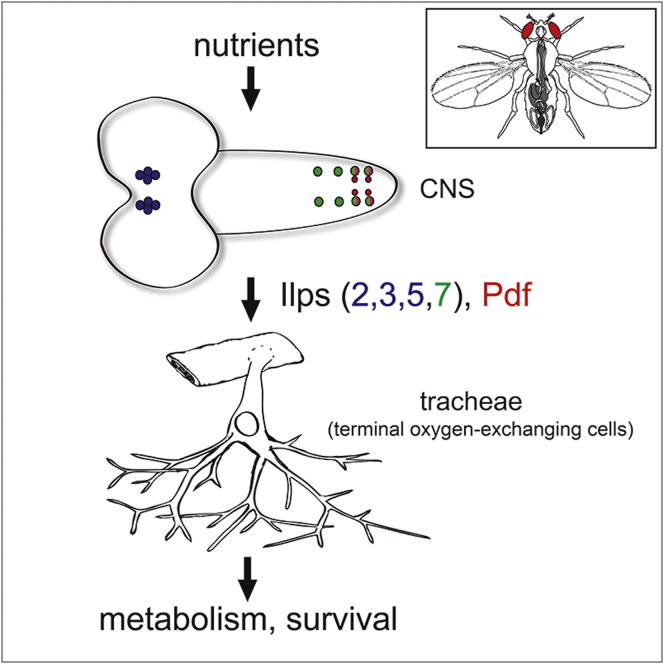

## Teaser

Previously unrecognized nutrient-dependent plasticity of the *Drosophila* tracheal system drives metabolic change and is regulated by distinct populations of nutrient- and oxygen-responsive neurons.

## Introduction

Unlike the more stereotypical development of the body’s main blood vessels, the formation of the capillary networks responsible for tissue perfusion is an adaptive process primarily governed by the metabolic needs of the target tissues (Fraisl et al., 2009, Potente et al., 2011). The plastic nature of this adaptive angiogenesis is further highlighted by the dramatic changes in vascularization observed in tumors or in obese adipose tissue: changes that contribute to the progression of pathologies such as cancer and obesity and are becoming increasingly central to their treatment (Cao, 2010, Kerbel, 2008, Lijnen, 2008). Although environmental factors such as diet are widely believed to affect the development and progression of these pathologies, exploration of the link between nutrition and angiogenesis has largely been confined to correlative studies. These include descriptions of the effects of gestational nutrition on the placental vasculature (Belkacemi et al., 2010, Rutland et al., 2007) or the pro/anti-angiogenic actions of nutrients and metabolites with a potential modulatory role in cancer (Adolphe et al., 2010, Kumar et al., 2013). A tantalizing new study has shown that increasing adipose tissue vascularization can ameliorate the deleterious metabolic effects of a high-fat diet, pointing to a central metabolic role for these vascular changes (Sung et al., 2013). However, whether modulation of angiogenesis is associated with metabolic benefits remains a controversial topic, partly because it is not trivial to genetically target the blood vessels of specific organs to recapitulate the changes associated with certain dietary interventions without affecting other cell types or vascular pools (Cao, 2010, Lijnen, 2008, Sun et al., 2012, Sung et al., 2013). Regardless of its metabolic consequences, adaptive angiogenesis is widely believed to be mechanistically driven by target-derived signals (Cao, 2007, Fraisl et al., 2009).

A close spatial association between mammalian nerves and vessels was observed as long ago as 1543 (Vesalius, 1543), an association that has subsequently been shown to result from mutual guidance or common pathfinding mechanisms during the formation of the neural and vascular networks (Carmeliet and Tessier-Lavigne, 2005, Mukouyama et al., 2005, Mukouyama et al., 2002, Quaegebeur et al., 2011). Notably, interplay of innervation and vascularisation of internal organs has also been described (Davies, 2009). A functional role for these neurovascular interactions was suggested following the discovery that vessel abnormalities precede a number of neurodevelopmental and neurodegenerative disorders: an observation that points to angiogenesis as a therapeutically relevant process (Quaegebeur et al., 2011, Storkebaum et al., 2011). The question remains whether, in a reciprocal process, neuronal activity may affect adaptive angiogenesis. In spite of some intriguing associations (Asano et al., 1997, Tonello et al., 1999), no neuronal populations have been identified that effect long-lasting changes in angiogenesis in response to environmental factors.

*Drosophila melanogaster* has an open circulation, but its tracheal system has a role analogous to that of the vertebrate vasculature in supplying tissues and internal organs with oxygen (Fraisl et al., 2009, Uv et al., 2003). During embryogenesis, developmental mechanisms akin to those discovered in the vertebrate lung and vasculature make use of signaling pathways such as fibroblast growth factor (FGF) signaling to sculpt this complex tracheal network of interconnected tubes (Ghabrial et al., 2003, Javerzat et al., 2002, Metzger et al., 2008, Uv et al., 2003). These embryonic proliferative and morphogenetic stages are superseded by a larval period of extensive, but mechanistically less understood, cellular growth. Growth is particularly prominent in the tracheal terminal cells: the cells at the end of each airway that make contact with target tissues and through which gas exchange takes place (Ghabrial et al., 2003, Uv et al., 2003). Like vertebrate capillaries, *Drosophila* tracheal terminal cells branch profusely in response to low oxygen using conserved FGF and hypoxia-inducible factor (HIF) signaling pathways (Centanin et al., 2008, Jarecki et al., 1999). This hypoxic remodeling has been assumed to be the only source of tracheal plasticity and, in normal conditions, the tracheal system is generally believed to grow in proportion to the whole organism. In this study, we use a combination of genetic approaches, metabolic profiling, and in vivo imaging to uncover previously unrecognized nutritional plasticity in the fly tracheal system. In contrast to the known target-derived mechanisms of adaptive remodeling, we find this plasticity to be regulated by a mechanism, previously undescribed in either flies or vertebrates, involving nutrient-responsive neurons effecting long-lasting and metabolically significant changes in tracheal architecture.

## Results

### Branching of Tracheal Terminal Cells Is Regulated in an Organ-Specific Fashion According to Both Previous and Current Nutrient Availability

**Figure 1:**
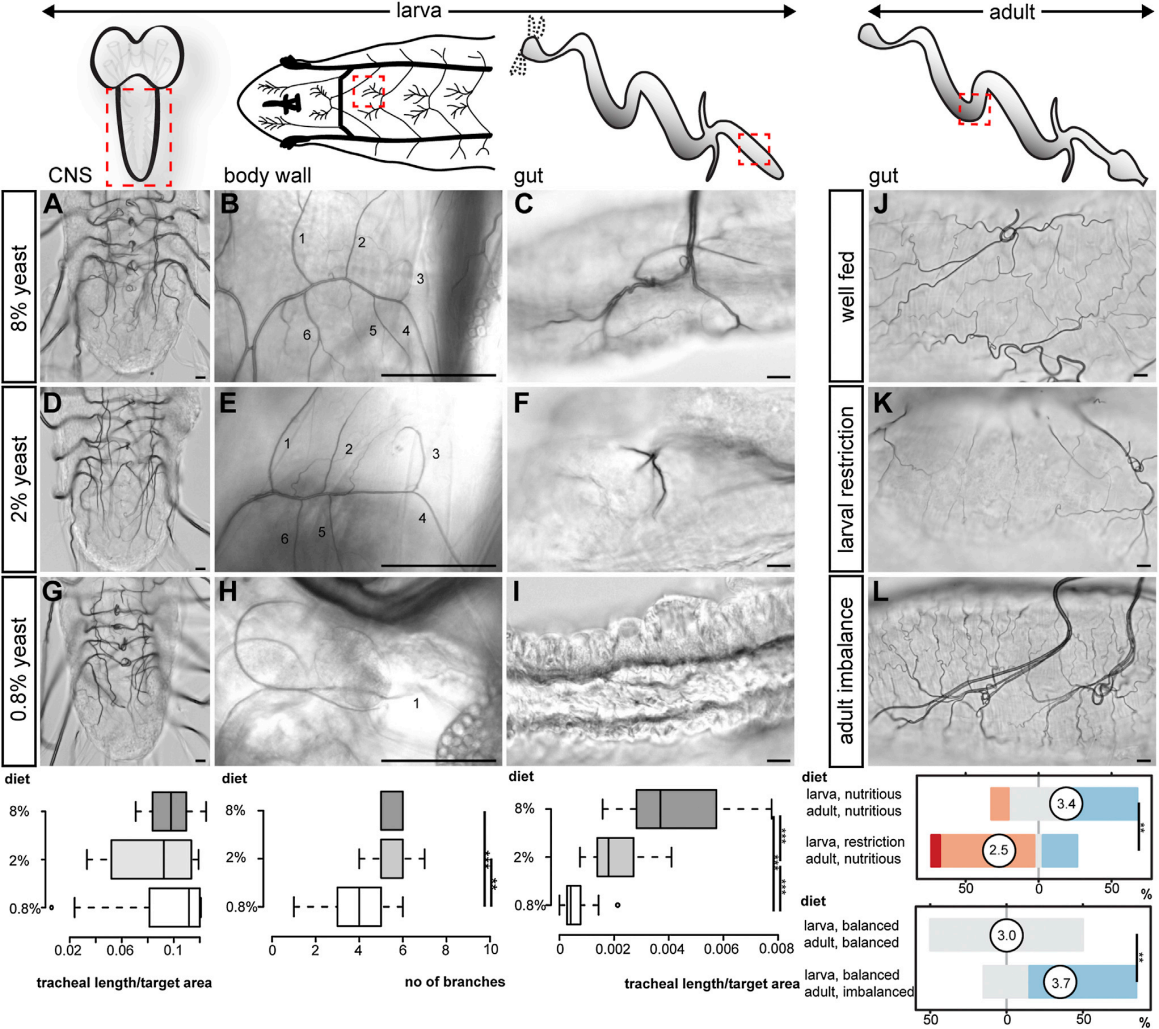
Nutritional and Organ-Specific Plasticity of Different Tracheal Subsets (A–C) Representative tracheation of the ventral nerve cord (VNC) (A), body wall (B), and gut (mid-hindgut, C) in well-fed larvae (8% yeast). (D–F) A mild nutrient restriction (2% yeast) does not affect CNS (D) or body wall (E) tracheae, but leads to reduced tracheal terminal growth in the gut (mid-hindgut, F). (G–I) Severe nutrient restriction (0.8% yeast) does not affect CNS tracheae (G), but leads to reduced coverage of both body wall (H) and gut (I, mid-hindgut). For body wall: p < 0.001 (8% versus 0.8%), p = 0.004 (2% versus 0.8%). For mid-hindgut: p < 0.0001 (8% versus 0.8%), p < 0.0001 (8% versus 2%), and p < 0.0001 (2% versus 0.8%). n = 10–24/set. (J) Representative gut tracheation (mid-midgut) of a 7-day-old adult fly reared on a nutritious (8% yeast) diet both during larval and adult stages. (K) Representative tracheation of the same intestinal region in an age-matched fly subject to an identical dietary regime as an adult, but exposed to a restricted diet (0.8% yeast) during larval life. Reduced branching is apparent. (L) Increased tracheation of the same region in a representative adult fly reared under standard conditions and exposed to 9% sucrose for 7 days. Quantifications of the adult phenotypes (J to L) are displayed below these panels. p = 0.001 (well-fed – larval restriction) and p < 0.0001 (balanced – adult imbalance), n = 17–33/set. Scale bars, 10 μm in all images except for (B), (E), and (H), 100 μm. See also Figures S1 and S2. Color coding for this and subsequent Likert levels are displayed as follows: red (strongly reduced), orange (reduced), gray (unchanged), light blue (increased), and dark blue (strongly increased). The mean (circled) is also displayed. See Experimental Procedures for additional information.

**Figure S1:**
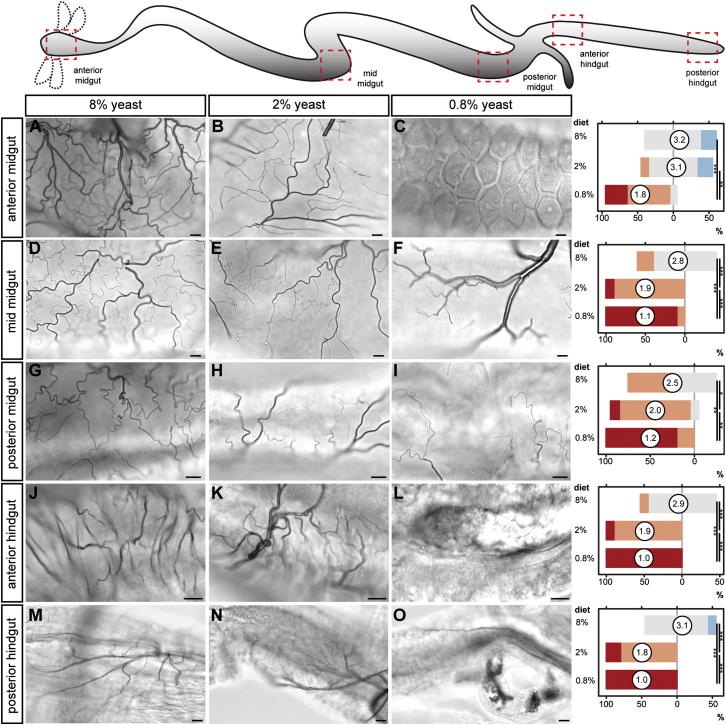
Nutritional Regulation of Different Intestinal Tracheal Subsets, Related to Figure 1 (A) Representative tracheation of the gut portions boxed in the cartoon: anterior midgut (A-C), mid-midgut (D-F), posterior midgut (G-I), anterior hindgut (J-L) and posterior hindgut (M-O) under the same three nutritional conditions described in in Figure 1. Quantifications for each intestinal portion are shown at the end of each row. For anterior midgut: p < 0.001 (8% versus 0.8%) and p = 0.001 (2% versus 0.8%), n = 10/set. For mid-midgut: p < 0.001 (8% versus 0.8%) and p < 0.001 (8% versus 2%) and p < 0.001 (2% versus 0.8%), n = 10/set. For posterior midgut: p = 0.001 (8% versus 0.8%), p = 0.047 (8% versus 2%), and p = 0.004 (2% versus 0.8%), n = 10/set. For anterior hindgut: p < 0.0001 (8% versus 0.8%), p < 0.001 (8% versus 2%), p < 0.001 (2% versus 0.8%), n = 10/set. For posterior hindgut: p < 0.0001 (8% versus 0.8%), p < 0.0001 (8% versus 2%), p < 0.001 (2% versus 0.8%), n = 10/set. Scale bars, 10 μm.

**Figure S2:**
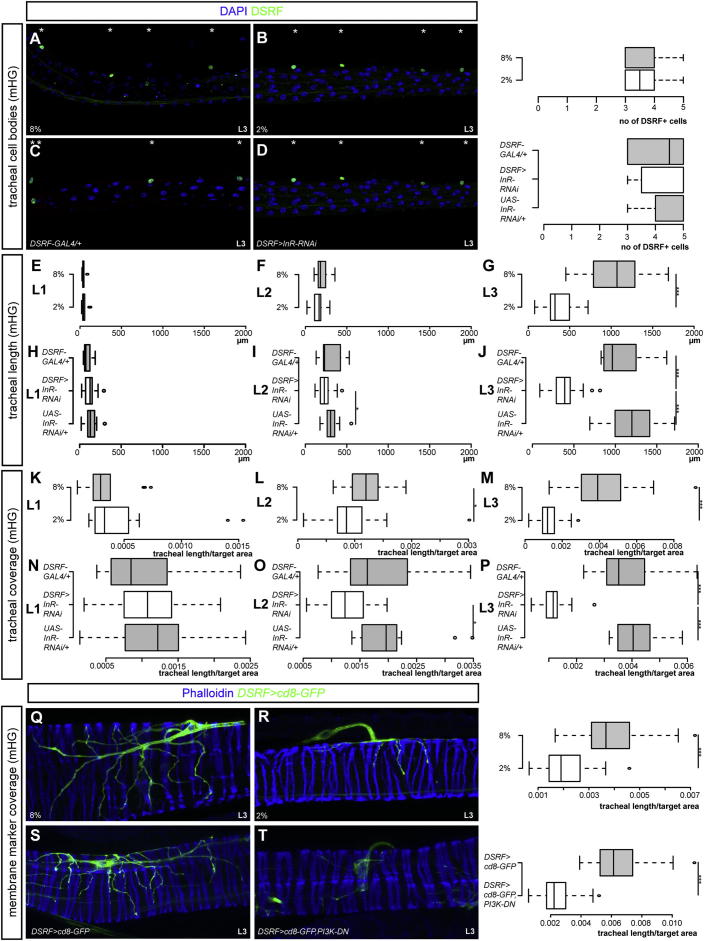
Cellular Analysis of the Reduced Tracheal Coverage Caused by Nutrient Restriction or Reduced Insulin Signaling, Related to Figures 1 and 2 (A to D) Staining with an anti-DSRF antibody that labels the tracheal terminal cell nuclei indicates that these cells are present in 3^rd^-instar larvae exposed to a mild nutrient restriction (B, not significantly different from the tracheal terminal cell number of well-fed larvae (A). See also quantifications to the right of (A) and (B). This rules out tracheal terminal cell death as the reason for the reduced intestinal tracheal coverage in nutrient-restricted larvae. Similarly, no differences in tracheal terminal cell number are apparent upon reduction of insulin signaling in *DSRF>InR-RNAi* larvae of the same stage (D, not significantly different from the tracheal terminal cell number of *GAL4* (C) or *UAS* controls (data not shown). See also quantifications to the right of C and D. In all panels, DAPI is used in blue to highlight the gut tissue, and asterisks are used above the tracheal terminal nuclei to highlight them. All panels show the mid-hindgut but the same results were obtained in other intestinal regions (data not shown). (E to P) Quantifications of the total length of tracheal arbours (E to J) and the ratio between this length and the gut area covered by the tracheal arbours (K to P) in the mid-hindgut of well-fed (8% yeast) versus nutrient-restricted (2% yeast) 1^st^-, 2^nd^- and 3^rd^-instar *OreR* larvae (E to G and K to M) or *DSRF>InR-RNAi* larvae versus *GAL4* and *UAS* controls (H to J and N to P). Tracheal coverage (as quantified by the length/area ratio in K to M for each larval instar) is mildly reduced in 2^nd^-instar larvae that have been nutrient restricted (L, p = 0.02), becoming strongly reduced by the 3^rd^-instar stage (M, p < 0.0001). However, the total length of these tracheal arbours increases with each larval instar (E to G), although not at the same rate as in well-fed larvae (G, p < 0.0001). n = 14–20/set. This suggests that the reduced tracheal coverage results from slower growth of these tracheal arbours. The same is true for the *DSRF>InR-RNAi* larvae (H to J and N to P). For tracheal length: in I, p = 0.02 versus *UAS* control but not significant versus *GAL4* control, and in J, p < 0.0001 versus either *GAL4* or *UAS* controls. For tracheal coverage: in O, p < 0.001 versus *UAS* control but not significant versus *GAL4* control, and in P, p < 0.0001 versus either *GAL4* or *UAS* controls. n = 13-18/set. (Q and R) Morphology of the tracheal terminal cell arbours of well-fed (Q, 8% yeast) or nutrient-restricted (R, 2% yeast) 3^rd^-instar larvae expressing the membrane-tagged reporter cd8-GFP from *DSRF-GAL4*. As the panels and quantification to the right of the panels show, tracheal coverage is reduced in nutrient-restricted 3^rd^-instar larvae (p < 0.0001, n = 26-29/set). This further confirms that the phenotype observed with DIC optics (an imaging technique best suited to the analysis of gas-filled tracheae), results, at least partly, from reduced branching rather than gas filling. (S and T) The same is true for *DSRF > cd8-GFP, PI3K-DN* (p < 0.0001 versus *DSRF>cd8-GFP* control, n = 22-25/set).

While subjecting wild-type *Drosophila* larvae to different dietary conditions, we observed that a severe reduction in dietary yeast (the main source of lipid and amino acids in the larval diet) was accompanied by an almost ubiquitous reduction in tracheal terminal cell branching, even when controlling for overall developmental delay by allowing nutrient-restricted larvae to develop to a comparable stage (Figures 1A–1C, 1G–1I and Figures S1A, S1C, S1D, S1F, S1G, S1I, S1J, S1L, S1M, and S1O available online). The single exception was the tracheal branches of the central nervous system (CNS), which were refractory to this dietary manipulation (Figures 1A and 1G). By contrast, a mild reduction in dietary yeast neither affected developmental timing nor led to major changes in the size of organs or that of most tracheal terminal cells (Figures 1D and 1E and data not shown) but did lead to a severe reduction in tracheal coverage throughout the digestive tract (Figures 1F, S1B, S1E, S1H, S1K, and S1N). Reduced tracheal coverage was not caused by cell death (Figures S2A and S2B) and could not be solely accounted for by defects in gas filling (Figures S2Q and S2R). Instead, it resulted from reduced tracheal terminal cell branching (Figures S2E–S2G, S2K–S2M, and S2Q–S2R). To investigate the reversibility of the tracheal changes described above, we reared larvae under the mild nutrient restriction conditions shown to reduce intestinal tracheation and transferred them to more nutritious food immediately after eclosion. Even after 7 days on a nutritious diet, the intestinal tracheae of these adults flies were significantly less branched than those of control adult flies always reared on a nutritious diet (Figures 1J and 1K), indicating that a defined period of nutrient restriction has long-term effects on the tracheal scaffold.

Dietary plasticity could be a feature unique to larval tracheae, given that their branches are undergoing extensive growth. To investigate whether adult tracheae are also responsive to diet, we allowed wild-type flies to develop under our standard nutritional conditions and then exposed them to nutritionally poor or imbalanced diets as adult flies. As Figure 1L shows, a 7 day nutritional imbalance (9% sucrose) led to increased intestinal tracheation of the mid-midgut, confirming the dietary plasticity of the tracheal system also in adult flies.

Collectively, these data uncover previously unrecognized nutritional plasticity of the insect tracheal system, shaped by both previous and current nutritional states. The tracheae of different organs exhibit different degrees of nutritional plasticity; intestinal tracheal terminal cells are particularly sensitive to a reduction in yeast availability, while CNS tracheae are preferentially spared.

### Differential, Cell-Autonomous Activation of Insulin Signaling Mediates Tracheal Terminal Cell Growth and Underlies the Enhanced Plasticity of Intestinal Tracheae

**Figure 2:**
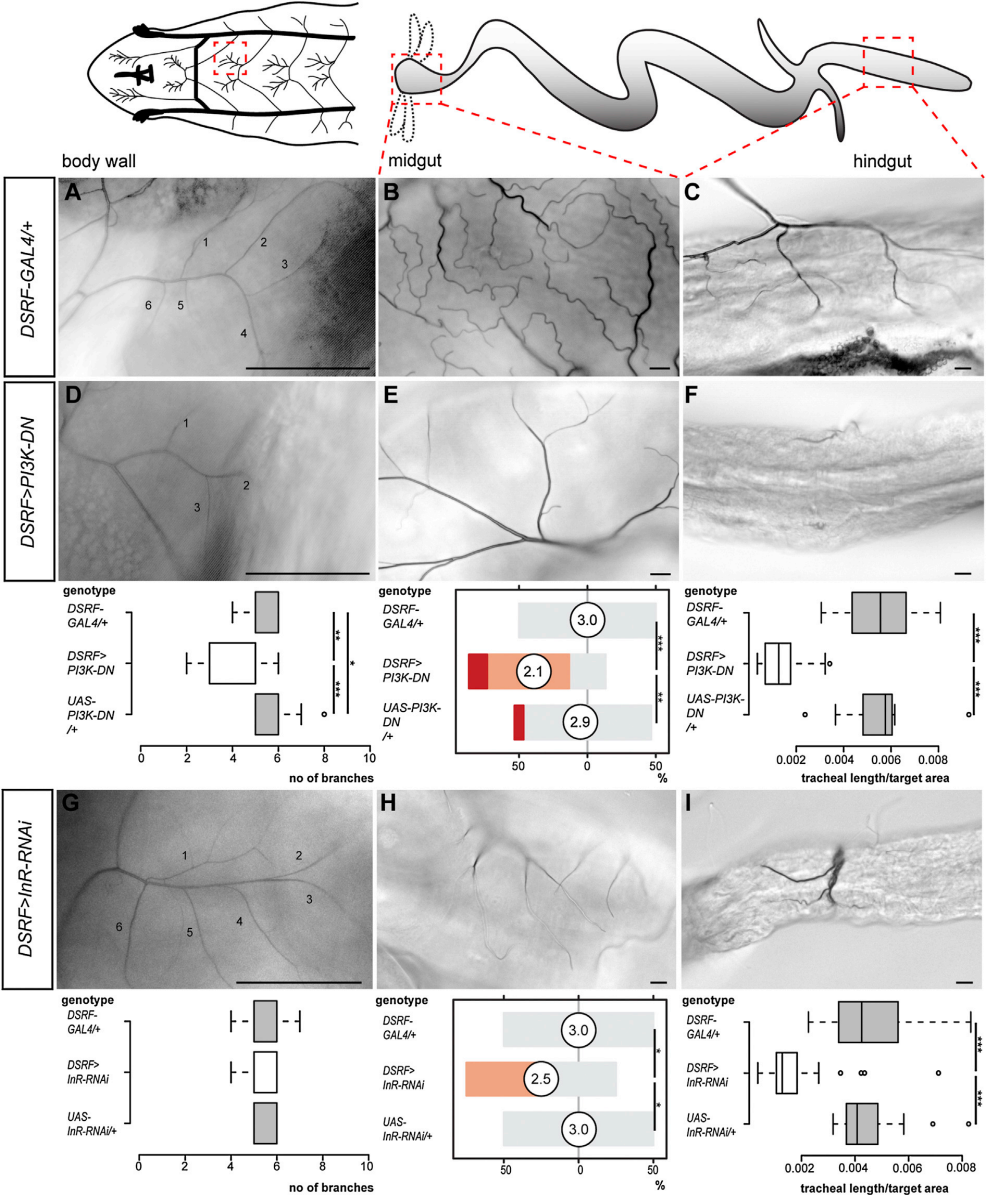
Organ-Specific Effects of Reduced Insulin Signaling on Tracheal Coverage (A–C) Representative tracheation of the areas boxed in the cartoons in control larvae: body wall (A), midgut (anterior, B), and hindgut (mid-hindgut, C). (D–F) Expression of PI3K-DN in tracheal terminal cells leads to reduced branching in body wall (D), midgut (anterior, E), and hindgut (mid-hindgut, F). For body wall: p = 0.001 (*DSRF>PI3K-DN* versus *GAL4* control), p < 0.001 (*DSRF>PI3K-DN* versus *UAS* control), p = 0.03 (*GAL4* versus *UAS* control), n = 13–15/set. For anterior midgut: p < 0.0001 (*DSRF>PI3K-DN* versus *GAL4* control), p < 0.001 (*DSRF>PI3K-DN* versus *UAS* control), n = 15/set. For mid-hindgut: p < 0.0001 (*DSRF>PI3K-DN* versus *GAL4* control), p < 0.0001 (*DSRF>PI3K-DN* versus *UAS* control), n = 19–25/set. (G–I) Driving RNAi against *InR* from the same driver line does not affect body wall tracheae (G) but leads to reduced branching in the midgut (anterior, H) and hindgut (mid-hindgut, I). Scale bars, 10 μm in all images except for (A), (D), and (G), 100 μm. For body wall: n = 15–20/set. For anterior midgut: p = 0.014 (*DSRF>InR-RNAi* versus *GAL4* control), p = 0.014 (*DSRF>InR-RNAi* versus *UAS* control), n = 10/set. For mid-hindgut: p < 0.0001 (*DSRF>InR-RNAi* versus *GAL4* control), p < 0.0001 (*DSRF>InR-RNAi* versus *UAS* control), n = 27–28/set. See also Figures S2 and S3.

**Figure S3:**
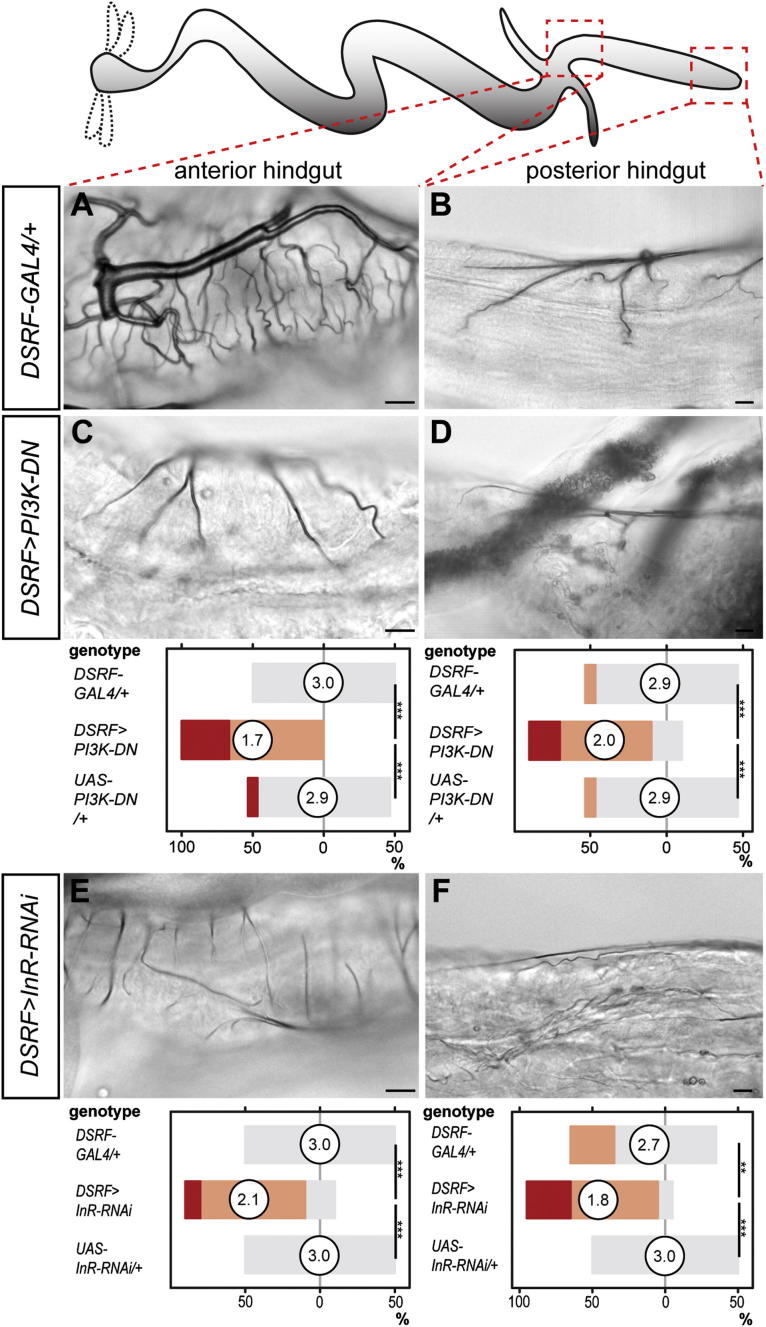
Effects of Reduced Insulin Signaling in the Tracheal Terminal Cells of Additional Intestinal Portions, Related to Figure 2 Representative tracheation of the areas boxed in the cartoons in control flies: anterior hindgut (A) and mid-hindgut (B). Expression of PI3K-DN specifically in tracheal terminal cells results in reduced branching in the anterior (C) and posterior hindgut (D). Tracheal branching quantifications for controls and *DSRF>PI3K-DN* larvae are shown separately for each tracheal subset below each column. p < 0.0001 (*DSRF>PI3K-DN* versus *GAL4* control) and p < 0.0001 (*DSRF>PI3K-DN* versus *UAS* control) for both anterior and posterior hindgut, n = 15/set. Driving RNAi against *InR* from the same driver line leads to reduced branching in these same gut portions: anterior hindgut (E) and posterior hindgut (F). Tracheal branching quantification for controls and *DSRF>InR-RNAi* larvae are shown separately for each tracheal subset below each panel. For anterior hindgut: p < 0.001 (*DSRF>InR-RNAi* versus *GAL4* control), p < 0.001 (*DSRF>InR-RNAi* versus *UAS* control). For posterior hindgut: p = 0.005 (*DSRF>InR-RNAi* versus *GAL4* control), p < 0.001 (*DSRF>InR-RNAi* versus *UAS* control). n = 10 flies/set. Scale bars, 10 μm.

We then focused on the larval phenotypes to investigate the molecular mechanisms of nutritional plasticity. Hypoxia, the only known regulator of tracheal plasticity, has been shown to promote tracheal branching by inducing FGF ligand in target tissues and receptor upregulation in tracheal cells (Centanin et al., 2008, Jarecki et al., 1999). Although downregulation of the FGF receptor gene *Breathless* (*Btl*) did lead to reduced tracheation in most scored tissues, consistent with the known FGF requirement for the establishment of the tracheal scaffold during earlier developmental stages (Ghabrial et al., 2003, Uv et al., 2003), further attempts to manipulate FGF signaling or to detect FGF ligand expression and differential pathway activation under different nutritional conditions all failed to support a role for FGF signaling in coupling nutrition with larval tracheal growth (data not shown). These included expression of Btl, constitutively active Btl, and its ligand Branchless (Bnl) in tracheal terminal cells and analysis of Bnl and Stumps (a downstream signaling component) expression under different nutritional conditions. We then turned our attention to the insulin signaling pathway: the major coordinator of nutrient intake and tissue size in all animals including *Drosophila* (Andersen et al., 2013). We first suppressed the intracellular insulin signal transducer phosphoinositide 3-kinase (PI3K) by expressing the dominant-negative Dp110^D954A^ (referred to as PI3K-DN; Leevers et al., 1996) in tracheal terminal cells using *DSRF-GAL4* (Gervais and Casanova, 2011). This led to reduced tracheal terminal cell branching both in the periphery and throughout the digestive tract, but not in the CNS (Figures 2A–2F, S2S, S2T, S3A–S3D and data not shown): a reduction qualitatively and quantitatively comparable to that observed in these tracheal terminal cells following severe nutrient restriction (Figures 1G–1I). As in the case of diet, the intestinal branches appeared to be more severely affected by this manipulation. Because the selective intestinal phenotype was not caused by stronger GAL4 expression in intestinal tracheae (data not shown), we tested whether it resulted from increased sensitivity to insulin signaling. To this end, we made use of an RNAi construct against the insulin receptor (InR) known to lead to incomplete receptor downregulation and a milder reduction in insulin signaling (Slaidina et al., 2009, Willecke et al., 2011). Driving this RNAi transgene in all tracheal terminal cells led to a significant reduction in intestinal, but not body wall or CNS, tracheal coverage (Figures 2G–2I, S3E, and S3F and data not shown). As in the case of dietary or PI3K manipulations, reduced coverage resulted from reduced tracheal terminal cell branching (Figures S2C, S2D, S2H–S2J, and S2N–S2P).

Together, these results confirm the cell-autonomous role for the insulin signaling pathway in the regulation of tracheal terminal cell growth and suggest that the enhanced nutritional plasticity of the gut tracheae is a consequence of their higher sensitivity to insulin signaling.

### Different Tracheal Subsets are Combinatorially Modulated by Both Systemic and Local Insulin- and VIP-like Neuropeptides

**Figure 3:**
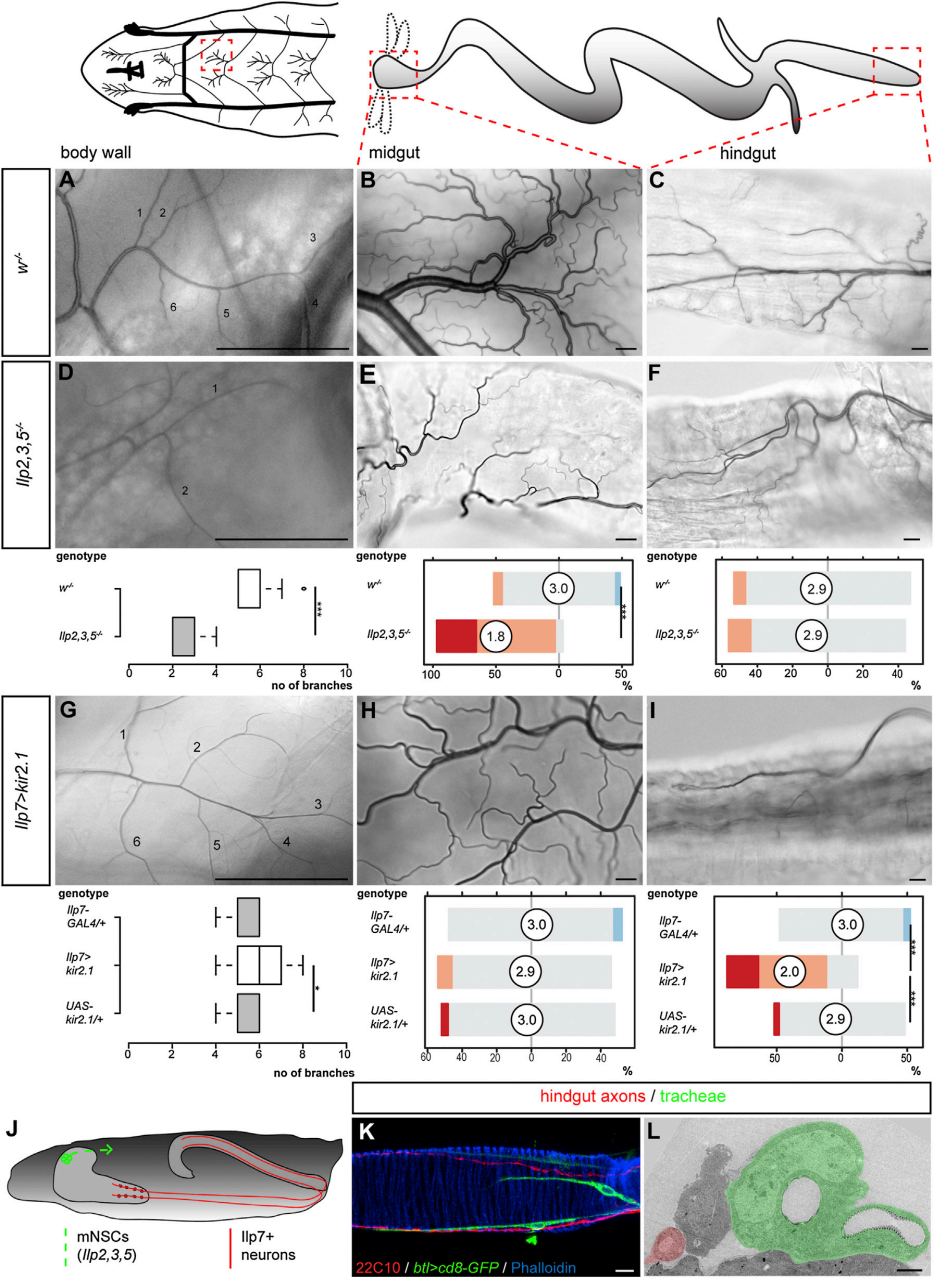
Two Subsets of Insulin-Producing Neurons Regulate the Growth of Different Tracheal Subsets (A–C) Representative terminal tracheation in well-fed control larvae. The specific body wall/gut areas are boxed in the cartoons: body wall (A), midgut (B, anterior), and hindgut (C, posterior). (D–F) Reduced branching is apparent in equivalent areas of the body wall (D), midgut (E), but not hindgut (F) in well-fed and genetically matched *Ilp2,3,5* mutants. p < 0.0001 for both body wall and anterior midgut. n = 16–35/set. (G–I) Representative terminal tracheation in the same body regions of well-fed control larvae upon silencing of the hindgut-innervating Ilp7 neurons. No effect is apparent in body wall (G) or anterior midgut (H), but the tracheal branching in the posterior hindgut is significantly reduced (I). For body wall: p = 0.048 (*Ilp7 > kir2.1* versus *UAS* control), but not significant versus *GAL4* control. For posterior hindgut: p < 0.0001 (*Ilp7 > kir2.1* versus *GAL4* control) and p < 0.0001 (*Ilp7 > kir2.1* versus *UAS* control). n = 12–18/set for body wall, 22–27/set for guts. (J) Larval neuroanatomy of the two subsets of insulin-producing neurons: Ilp2, Ilp3, and Ilp5 (in green) are released from the brain mNSCs into the circulation. Ilp7-producing neurons located in the posterior segments of the VNC (in red) send long axons that exit in the posterior nerves that innervate both sides of the hindgut. (K) The two hindgut nerves (labeled in red with the broad neuronal marker 22C10) are found in close proximity to the posterior visceral tracheal branches in the posterior hindgut of a 1^st^-instar larva (visualized using a membrane-tagged GFP expressed from the pan-tracheal driver *btl-GAL4)*. Phalloidin (in blue) was used to highlight the visceral muscles. (L) Transmission electron microscopy of a posterior hindgut cross-section highlighting the proximity between the hindgut nerve axons (highlighted in red) and tracheae (in green). Scale bars, 10 μm in all images except for (A), (D), and (G), 100 μm and (L), 2,000 nm. See also Figure S4.

**Figure S4:**
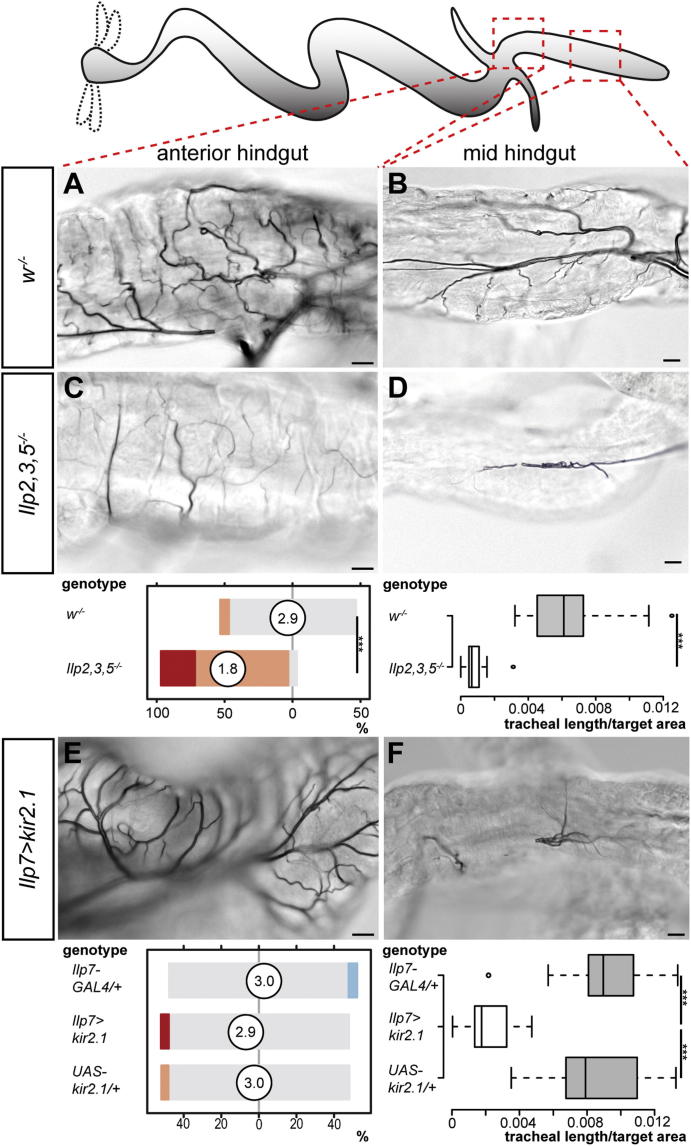
Differential Regulation of Hindgut Tracheae by the Two Subsets of Insulin-Producing Neurons, Related to Figure 3 (A–B) Representative terminal tracheation in well-fed control larvae. The specific gut areas are boxed in the cartoons: anterior hindgut (A) and mid-hindgut (B). (C–D) Reduced branching is apparent in equivalent areas in well-fed and genetically matched *Ilp2,3,5* mutants. Quantifications for each tracheal subset are shown below each column. p < 0.0001 for both anterior hindgut and mid-hindgut, n = 15–31/set. (E–F) Representative terminal tracheation in the same body regions of well-fed larvae upon silencing of the hindgut-innervating Ilp7 neurons. No effect is apparent in the anterior hindgut (E), but the tracheal branching in the mid-hindgut is significantly reduced (F). For mid-hindgut: p < 0.0001 (*Ilp7 > kir2.1* versus *GAL4* control), p < 0.0001 (*Ilp7 > kir2.1* versus *UAS* control), n = 17–27/set for both anterior and mid-hindgut. Scale bars, 10 μm.

In *Drosophila* larvae, nutrient restriction leads to growth inhibition, caused by the reduced release of several insulin-like peptides (Ilps) from brain insulin-producing cells (the so-called median neurosecretory cells, mNSCs, represented schematically in Figure 3J) into the hemolymph (Géminard et al., 2009). A triple mutation of the three main mNSC Ilps (*Ilp2*, *Ilp3*, and *Ilp5*; Grönke et al., 2010) largely recapitulated the phenotype resulting from expression of PI3K-DN in tracheal terminal cells. Indeed, reduced growth was observed in both body wall (Figures 3A and 3D) and intestinal tracheal terminal cells (Figures 3B, 3E, and S4A–S4D). However, we found the posterior hindgut tracheal branches to be spared in these larvae (Figures 3C and 3F). Immunohistochemical and ultrastructural analyses of this intestinal portion revealed that these posterior tracheal branches were adjacent to the two hindgut nerves that run along both sides of the hindgut (Figures 3K, 3L, and 7B). We have previously shown that axons emanating from a different population of CNS Ilp-producing neurons, the Ilp7 neurons, contribute to this innervation (Figure 3J; Miguel-Aliaga et al., 2008) and thus could provide a local peptide supply to this portion of the gut. Functional inactivation of the Ilp7 neurons by expression of the inward-rectifying potassium channel kir2.1 or by expression of tetanus toxin light chain did not affect most tracheae but led to reduced tracheal coverage of two portions of the hindgut (Figures 3G–3I, S4E, and S4F and data not shown): the posterior hindgut (Figures 3C and 3I), where the Ilp7 axons are adjacent to the posterior visceral tracheal branches (Figures 3K and 7B), but also the mid-hindgut (Figure S4F), which we had also found to be regulated by systemic mNSC-derived Ilps (Figures S4B and S4D). In this latter region, the visceral tracheal branches emanate from the segmentally repeated main lateral branches (Figures 7A and 7B) and do not abut the Ilp7 axons, suggesting paracrine growth regulation.

**Figure 4:**
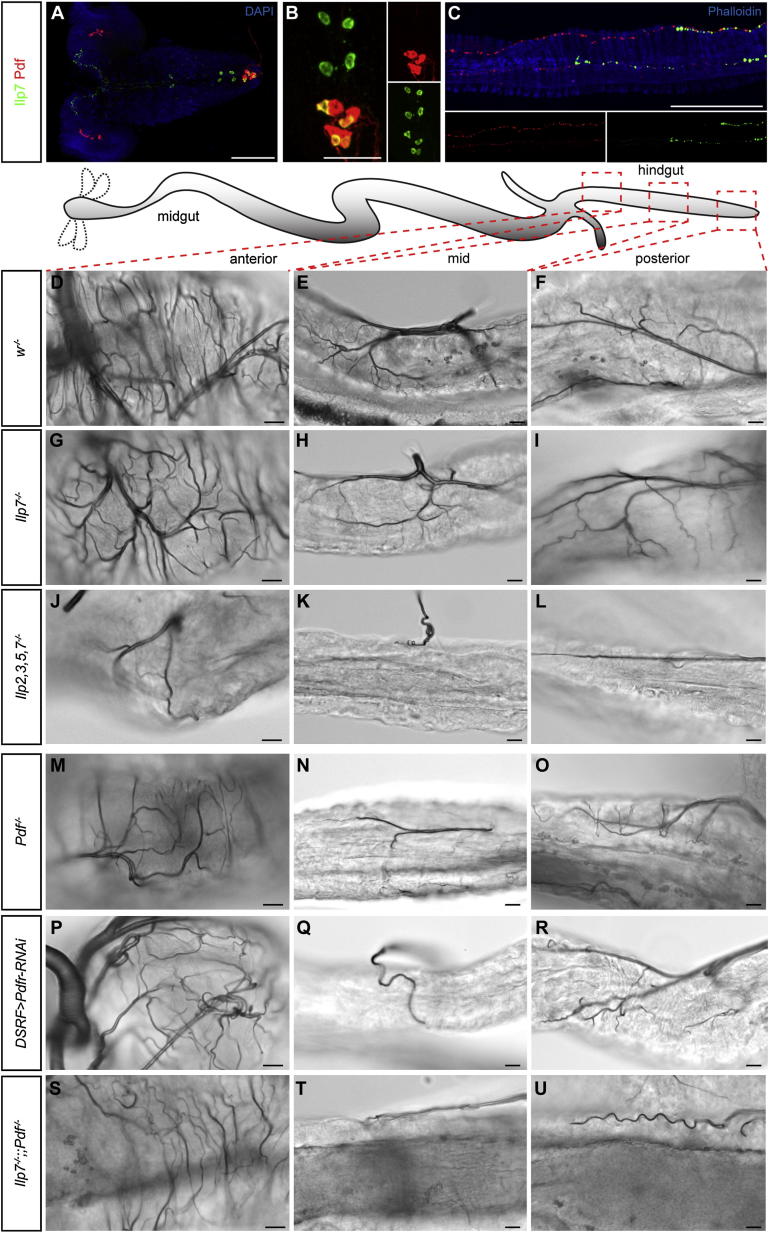
Regional Regulation of Intestinal Tracheae by Multiple Ilp and Pdf Neuropeptides (A) Expression of Ilp7 (green) and Pdf (red) neuropeptides in a 1^st^-instar VNC. Note the cell bodies in the posterior-most segments (to the right). DAPI (in blue) was used to visualize the CNS. Anterior is to the left. (B) Higher magnification image of these posterior cell bodies: four of the eight Ilp7-expressing neurons (those located in the two posterior-most segments) coexpress Pdf. Pdf is also expressed by four additional neurons in these segments. Anterior is to the top. (C) Regional expression of the Ilp7 and Pdf peptides produced by the neurons in (B) in a 2^nd^-instar hindgut. Anterior is to the left, and the visceral muscles are highlighted in blue with phalloidin. Both peptides are present in varicosities along the hindgut nerves, but the anterior-most nerve endings are only positive for Pdf. (D–F) Representative hindgut tracheation in well-fed control larvae. The specific gut regions are boxed in the cartoons: anterior (D), mid- (E), and posterior hindgut (F). (G–I) *Ilp7* mutation does not affect branching in the anterior or posterior hindgut but results in mildly reduced branching in the mid-hindgut. (J–L) A severe reduction in branching is apparent in the entire hindgut of mutants lacking *Ilp7* as well the systemic *Ilp2*, *Ilp3*, and *Ilp5* peptides. (M–O) *Pdf* mutation does not affect branching in the anterior hindgut (M) or posterior hindgut (O) but leads to reduced tracheal growth in the mid-hindgut (N). (P–R) Downregulation of the Pdf receptor specifically in tracheal terminal cells using *DSRF-GAL4* does not affect branching in the anterior hindgut (P) or posterior hindgut (R) but leads to significantly reduced growth in the mid-hindgut (Q). (S–U) The intestinal tracheal coverage in double mutants lacking both Pdf and Ilp7 peptides is indistinguishable from that of control flies in the anterior hindgut (S), but it is strongly reduced in both the mid-hindgut (T) and posterior hindgut (U). See also Figure S5 for quantifications and Figure 7 for a summary of this regional regulation of tracheae by different peptides. Scale bars, 10 μm in all images except for (A), (B), and (C), 100 μm, 50 μm, and 100 μm, respectively.

**Figure S5:**
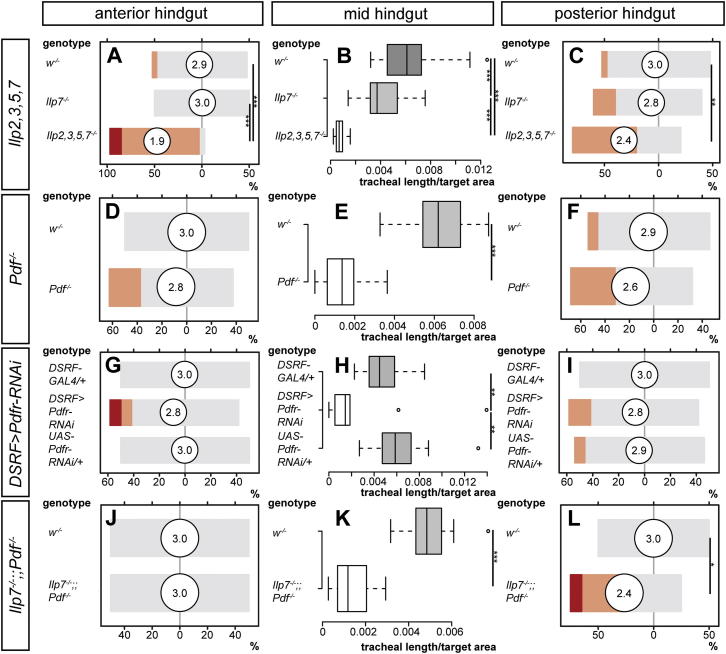
Quantifications of Phenotypes Resulting from *Ilp* and/or *Pdf* Mutation and from *Pdfr* Tracheae-Specific Downregulation, Related to Figure 4 (A–C) *Ilp7* mutation does not affect tracheal growth in anterior hindgut (A), or posterior hindgut (C) when compared to genetically matched control flies, but has a small effect on the tracheae of the mid-hindgut. By contrast, tracheal branching is significantly reduced in all three portions in quadruple mutants lacking *Ilp7* and mNSC-derived *Ilp2*, *Ilp3* and *Ilp5*. For anterior hindgut: p < 0.0001 (*Ilp2,3,5,7* mutants versus either *Ilp7* or *w* mutants). For mid-hindgut: p < 0.001 (*Ilp7* mutants versus *w*) and p < 0.0001 (*Ilp2,3,5,7* mutants versus either *Ilp7* or *w* mutants). For posterior hindgut: p = 0.002 (*Ilp2,3,5* mutants only versus *w* mutants). n = 10–23/set for all three gut regions. (D–F) The tracheal branching of *Pdf* mutants is not significantly different from genetically matched controls in the anterior hindgut (D) or posterior hindgut (F), but it is reduced in the mid-hindgut (E, p < 0.0001). n = 14–18/set for all three gut regions. (G–I) Downregulation of the Pdf receptor specifically in tracheal terminal cells using *DSRF-GAL4* does not affect branching in the anterior hindgut (G) or posterior hindgut (I), but leads to significantly reduced growth in the mid-hindgut (H, p = 0.005 (*DSRF>Pdfr-RNAi* versus *GAL4* control) and p = 0.002 (*DSRF>Pdfr-RNAi* versus *UAS* control). n = 10-15/set for all three gut regions. (J–L) The intestinal tracheal branching of double mutants lacking both Pdf and Ilp7 peptides is indistinguishable from that of control flies in the anterior hindgut (J), but it is reduced in both the mid-hindgut (K, p < 0.0001) and posterior hindgut (L, p = 0.014). n = 10–12 in all three gut regions.

We then characterized the peptidergic profile of the central neurons contributing to the hindgut nerves using immunohistochemistry. Four of the eight Ilp7-expressing neurons coexpress pigment dispersing factor (Pdf) (Figures 4A and 4B): a neuropeptide that shares functional and signaling similarities with vertebrate vasoactive intestinal polypeptide (VIP) (Taghert and Nitabach, 2012). Four other central hindgut-innervating neurons also express Pdf and bundle together with the Ilp7 hindgut nerves (Figure 4B; Talsma et al., 2012). Together, both neuronal populations deliver Pdf and Ilp7 to the hindgut in a regionalized fashion: Ilp7 is apparent only in the posterior hindgut, whereas Pdf is present in both posterior and mid-hindgut terminals (Figures 4C and 7B). Mutation of these peptides, alone or in combination, revealed complex control of different intestinal tracheal subsets by local Ilp7 and Pdf peptides in combination with the systemic Ilp2, Ilp3 and Ilp5 peptides (Figures 4D–4U, S5A–S5L, and 7B): in the posterior hindgut, neither loss of *Ilp7* alone nor *Ilp2*, *Ilp3*, and *Ilp5* together affected tracheal branching (Figures 4F, 4I, S5C, 3C, and 3F), but loss of all four peptides resulted in reduced tracheal terminal cell growth (Figures 4L and S5C), indicating partially redundant control of tracheal terminal growth. Loss of *Ilp7* or *Pdf* alone, or tracheal-specific downregulation of the *Pdf* receptor (*DSRF-GAL4, UAS-Pdfr-RNAi*), resulted in reduced tracheal growth only in the mid-hindgut (Figures 4G–4I, 4M–4R and S5A–S5I): a region also affected by the lack of systemic Ilps (Figures S4B and S4D) and not directly exposed to Ilp7 peptide (Figure 7B). Finally, mutants lacking both *Ilp7* and *Pdf* displayed reduced tracheal growth in both the mid-hindgut and posterior hindgut (Figures 4T, 4U, S5K, and S5L), indicating that Ilp7 and Pdf act redundantly in the posterior hindgut.

Collectively, neuropeptide mutation and tracheal receptor downregulation experiments indicate that growth of tracheal terminal cells is directly regulated by the nervous system. The systemically secreted Ilps act as virtually pan-tracheal regulators, but in some intestinal portions they synergize in a combinatorial—and sometimes partially redundant—manner with locally delivered Ilp and Pdf neuropeptides.

### Exposure to Nutrients and Reductions in Oxygen Availability Elicit Calcium Responses in the Gut-Innervating Ilp7/Pdf Neurons

**Figure 5:**
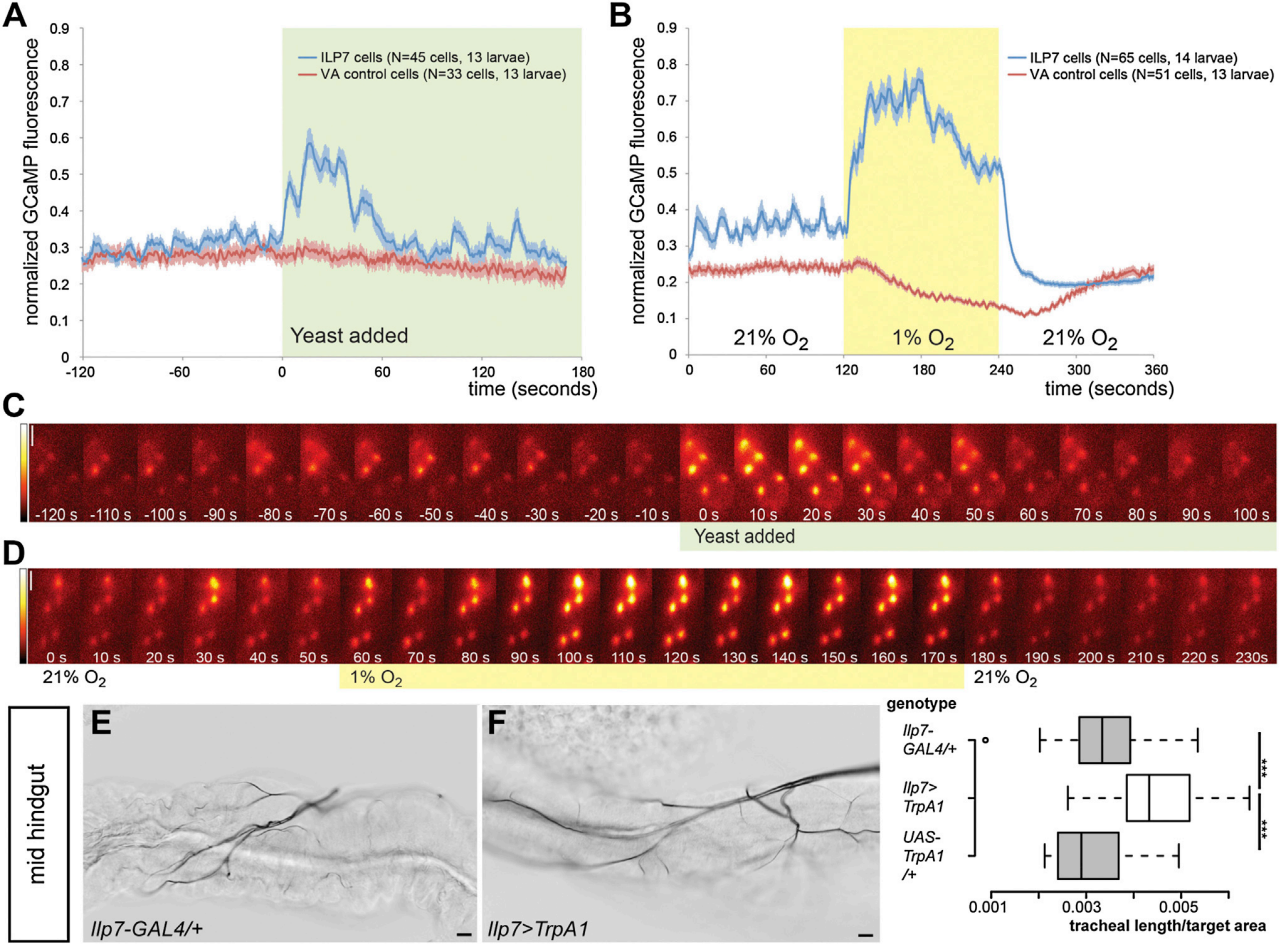
Regulation of Ilp7 Neuronal Activity by Nutrients and Hypoxia, and Its Effect on Tracheal Branching (A) Exposure to yeast leads to a transient Ca^2+^ rise in Ilp7 neurons. Activity returns to basal levels after one minute. No such response is observed in control Va neurons. (B) A switch from 21% to 1% ambient O_2_ elicits a rapid rise in Ca^2+^ in Ilp7 neurons that persists while O_2_ is low. Upon return to normoxia, the basal activity of the Ilp7 neurons is immediately abrogated. No Ca^2+^ rise is triggered in control Va neurons, which display a subtle drop in Ca^2+^ levels in response to hypoxia, as has previously been observed for different types of neurons in various species (Cheung et al., 2006, Fujiwara et al., 1987, Krnjević, 1999). Error bars denote SEM. (C and D) False color-coded single frames depicting GCaMP fluorescence in representative movies illustrating the response to yeast (C) or hypoxia (D) observed in Ilp7 neuronal cell bodies. Yellow/white indicates strong responses, red, low Ca^2+^ (false color scale is shown to the left). (E and F) 25°C thermogenetic activation of the TrpA1 channel expressed in Ilp7 neurons through larval development results in increased tracheal coverage of the midgut (F) relative to controls (E for *GAL4* control). Quantifications are displayed to the right of these two panels (p < 0.001 versus GAL4 control, p < 0.0001 versus UAS control, n = 23-27/set). Scale bars, 25 μm (C) and (D) or 10 μm (E) and (F). See also Figure S6.

**Figure S6:**
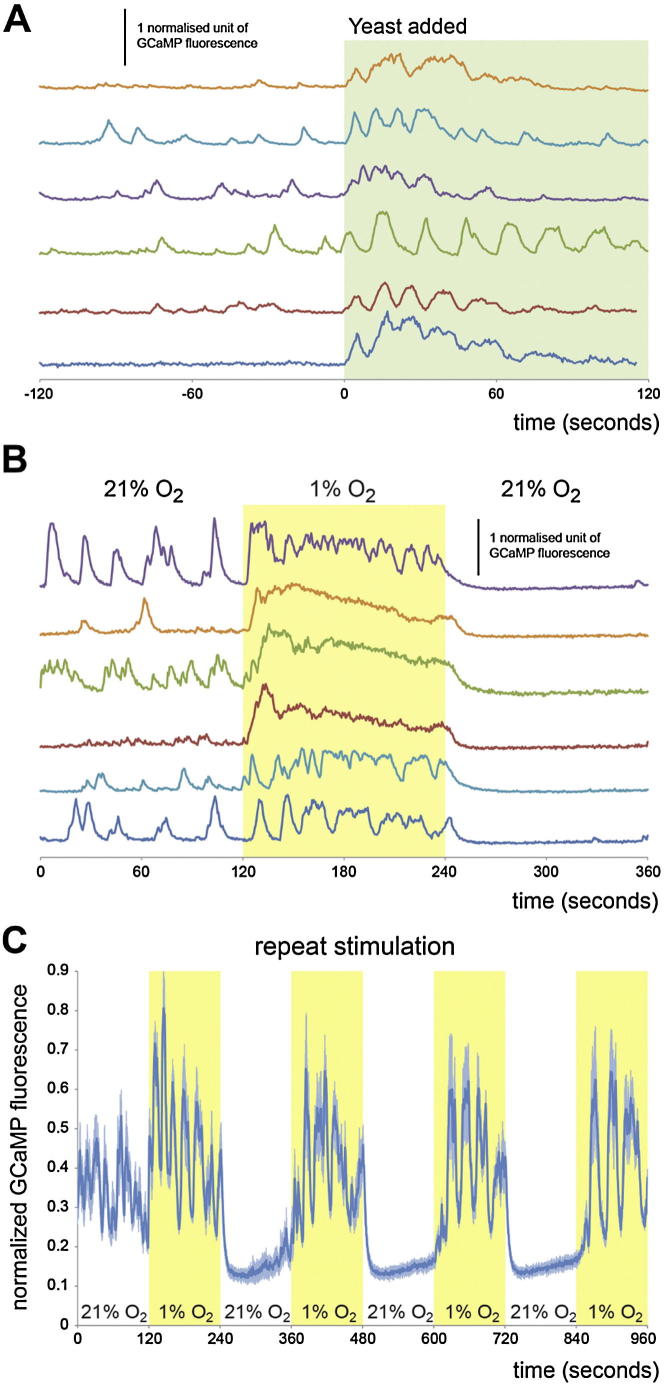
Dynamics of Ilp7 Neuronal Activation in Response to Nutrients and Hypoxia, Related to Figure 5 (A) Single-neuron traces for yeast-evoked Ca^2+^ responses in Ilp7 neurons. While most neurons respond by increasing the frequency and amplitude of Ca^2+^ spikes, a subset of them respond in a more tonic fashion. (B) Single-neuron traces for O_2_-evoked Ca^2+^ responses in Ilp7 neurons. Some neurons respond completely tonically, while others respond chiefly with Ca^2+^ transients. (C) Ilp7 neuron traces from a larva exposed to repeated 2 min cycles of 21% and 1% O_2_. Each repeat stimulation with 1% O_2_ causes a very similar response, indicating that the cells neither habituate to the hypoxic stimulus nor become unable to respond.

Both mNSCs and Ilp7 neurons have been shown to modulate feeding responses to nutrient scarcity in adult flies (Cognigni et al., 2011). However, the dietary dependency of Ilp release has only been investigated in mNSCs using immunohistochemistry (Géminard et al., 2009). To directly image neural activity in response to nutrients in vivo, we expressed the genetically encoded green fluorescent Ca^2+^ indicator GCaMP3 (Tian et al., 2009) in Ilp7 neurons, together with a red fluorescent protein to visualize the cell bodies. Ilp7 cell bodies displayed some transient activity in the absence of a stimulus, which rapidly increased following yeast presentation (Figures 5A, 5C, S6A, and Movie S1). In most neurons, the frequency and amplitude of the transient Ca^2+^ peaks increased and then adapted after about one minute, possibly a consequence of persistent exposure to yeast. This response was yeast-specific because exposure to sucrose did not elicit any responses in these neurons (data not shown), consistent with the yeast dependency of tracheal growth. It was also specific to Ilp7 neurons, given that GCaMP3 fluorescence intensity was unaffected by yeast in the Capa-expressing Va neurons, used as a control population of six unrelated peptidergic efferent neurons (Suska et al., 2011) (Figure 5A).

The only well-characterized environmental trigger of tracheal branching is hypoxia (Centanin et al., 2008, Jarecki et al., 1999). We therefore monitored oxygen-evoked Ca^2+^ responses in these two neuronal populations and found that hypoxia led to a fast and very robust response in the Ilp7—but not in the Va—neurons (Figures 5B, 5D, and S6B, and Movie S2). This response was qualitatively distinct from that resulting from yeast exposure. Indeed, it was predominantly tonic, although some animals mainly showed transient peaks of increased amplitude, and lasted throughout the hypoxic period, decreasing slightly over time. Interestingly, the return to normoxia almost completely abrogated the basal transient firing of Ilp7 neurons, suggesting hyperpolarization. This effect was not a consequence of excessive firing and cellular “exhaustion” because repeated hypoxic stimulation continued to activate the Ilp7 neurons (Figure S6C).

Together, these findings indicate that the activity of the Ilp7- and Pdf-producing neurons is increased in vivo by both nutritional cues and reductions in oxygen availability.

### Activation of the Ilp7/Pdf Neurons Promotes Tracheal Branching Locally

Together with previous Ilp/Pdf loss-of-function experiments, the above experiments suggested that nutritional modulation of Ilp neuronal activity underlies the nutritional plasticity of tracheae. To test this idea, we used thermogenetics to achieve persistent, low-level activation of the Ilp7 neurons throughout larval life by expressing the heat-sensitive channel TrpA1 from *Ilp7-GAL4* in larvae reared at 25°C. This promoted tracheal branching in a paracrine fashion; it increased branching of the adjacent visceral tracheal branch of the posterior hindgut and the tracheal terminal cells of the neighboring mid-hindgut, but did not redirect those of the anterior hindgut or other regions (Figures 5E and 5F, and data not shown). Hence, in addition to being necessary, Ilp7 neurons are sufficient to sustain tracheal growth in the hindgut.

### The Organ-Specific Modulation of Tracheation Is Metabolically, but Not Developmentally, Significant

**Figure 6:**
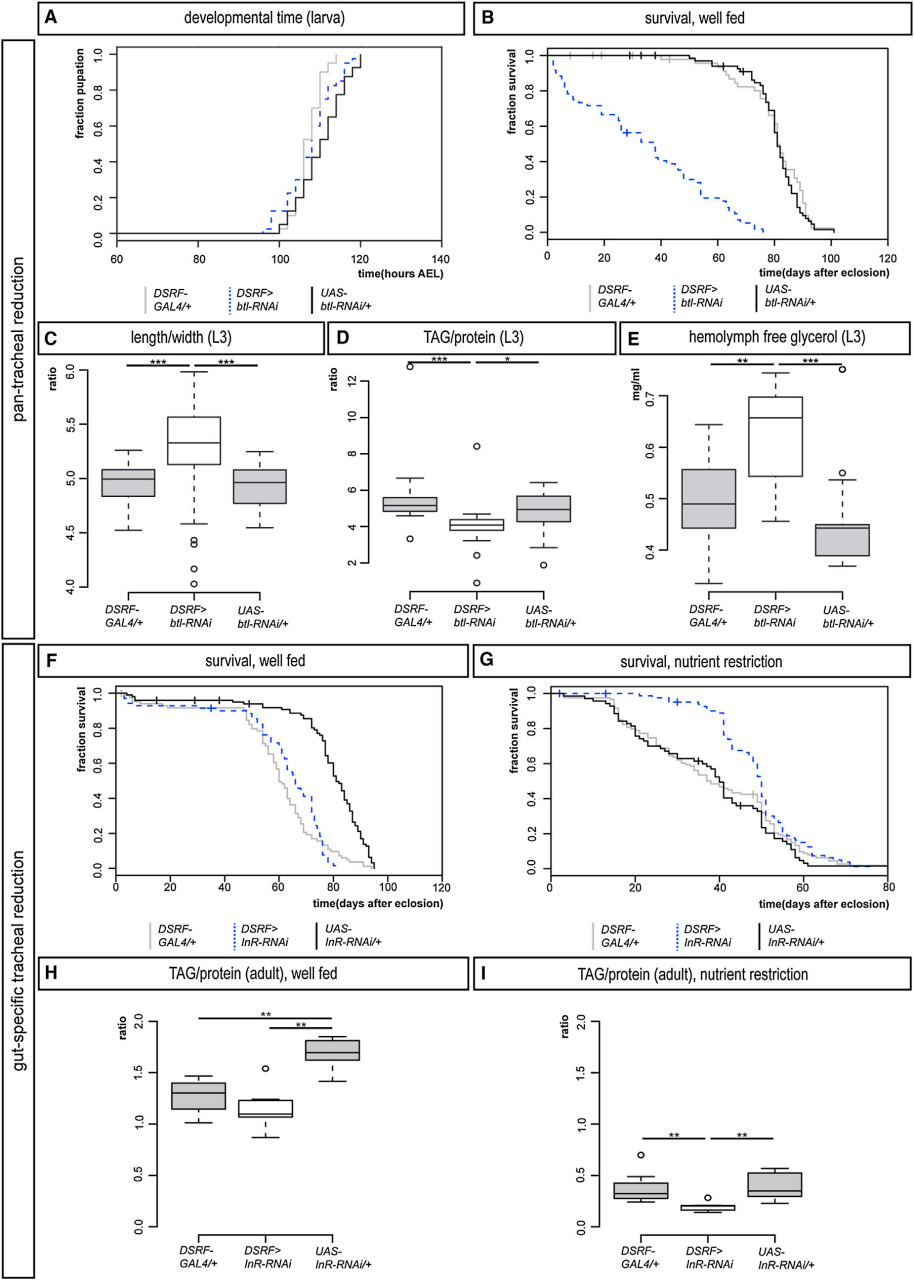
Distinct Effects on Energy Homeostasis Resulting from Pan-Tracheal or Gut-Specific Reductions in Tracheal Terminal Branching (A) Reduced growth of most tracheal terminal cells (achieved using *DSRF>btl-RNAi*) does not affect the time between egg laying and pupation (only the two controls are significantly different from one another, p < 0.001, n = 40 larvae/set). (B) This genetic manipulation leads to shorter-lived adult male flies in the presence of nutritious food (p < 0.0001 for all three comparisons, n = 70–120 flies/set). (C) *DSRF>btl-RNAi* larvae have an increased length to width ratio (p < 0.001 versus *GAL4* control, p < 0.0001 versus *UAS* control, n = 30 samples/set, total 300 larvae/set). (D) They also have a reduced fat/protein content ratio (p < 0.0001 versus *GAL4* control and p = 0.013 versus *UAS* control, n = 19 samples/set, total 190 larvae/set). (E) An increase in free glycerol is also apparent in their hemolymph (p = 0.002 versus *GAL4* control, p < 0.001 versus *UAS* control, n = 13 samples/set, total 130 larvae/set). (F) A gut-specific reduction in tracheal terminal cell growth (achieved using *DSRF>InR-RNAi*) does not affect the survival of adult male flies in well-fed conditions (n = 60–140 flies/set). (G) The same genetic manipulation leads to enhanced survival when adult male flies are subject to nutrient restriction (p < 0.0001 versus either control, p < 0.001 *GAL4* versus *UAS* controls, n = 110–120 flies/set). (H and I) The lipid stores of these adult males are relatively normal in well-fed conditions (H, p = 0.001 versus *UAS* control but not significant versus *GAL4* control, p = 0.002 *GAL4* versus *UAS* controls, n = 7 samples/set, total 70 flies/set), but they are more reduced than those of controls upon nutrient restriction (I, p = 0.002 versus either *UAS* or *GAL4* controls, n = 7 samples/set, total 70 flies/set). See also Figure S7.

**Figure S7:**
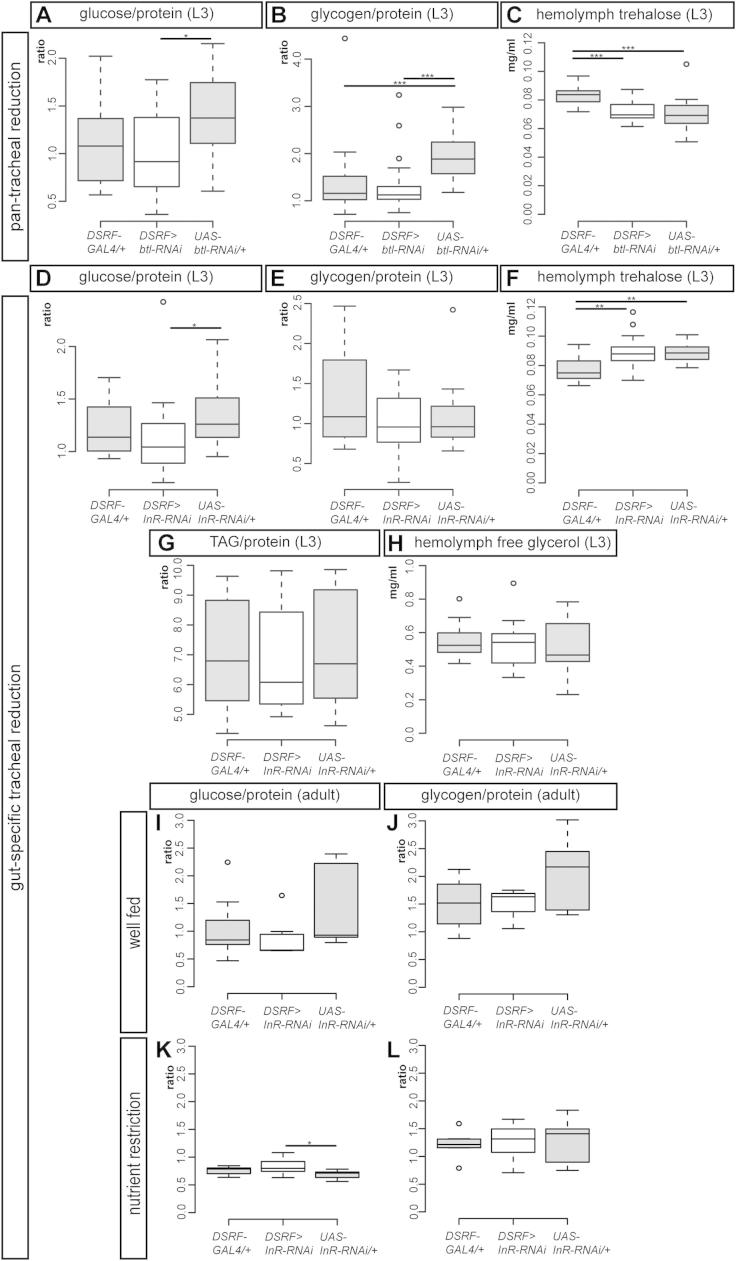
Additional Metabolic Quantifications Following Pan-Tracheal or Gut-Specific Reductions in Tracheal Terminal Branching, Related to Figure 6 (A) A reduction in the growth of most tracheal terminal cells (achieved using *DSRF>btl-RNAi*) does not affect the ratio between glucose and protein content (experimental flies are only marginally different from the *UAS* control, p = 0.011, n = 20/set). (B) The same manipulation does not affect the ratio between glycogen and protein content of whole 3^rd^-instar larvae (p < 0.0001 versus *UAS* control but not significant versus *GAL4* control, p < 0.001 *GAL4* control versus *UAS* control, n = 17/set). (C) The amount of trehalose in their hemolymph is also unaffected (p < 0.001 versus *GAL4* control but not significant versus *UAS* control, p < 0.0001 *GAL4* control versus *UAS* control, n = 17/set). (D–F) Reduction of tracheal terminal growth specifically in the gut (achieved using *DSRF>InR-RNAi*) has glycogen/protein ratio (E, n = 18/set) and hemolymph trehalose (F, p = 0.004 versus *GAL4* control but not significant versus *UAS* control, p = 0.002 *GAL4* control versus *UAS* control, n = 14/set). (G) Although a trend toward a reduced TAG/protein ratio is observed in this genotype, this ratio is not significantly different from that of control flies (n = 14/set). (H) The amount of free glycerol in their hemolymph is also unaffected (n = 15/set). (I–L) As adults, these flies have glucose/protein (I and K) and glycogen/protein (J and L) ratios comparable to those of controls, both in well-fed (I and J) and nutrient-restricted (K and L) conditions (for K, p = 0.04 versus *UAS* control but not significant versus *GAL4* control, n = 7/set).

The finding that tracheal branching is directly regulated by nutrient-responsive neurons suggests that tracheal terminal cells may be used by the nervous system as effectors of metabolic adaptations to nutrient availability. To investigate this possibility, we recapitulated the differential effects of nutrient restriction on tracheae by either reducing tracheal terminal cell growth in all tissues (except for the CNS tracheae, using *DSRF>btl-RNAi*), or specifically in the gut tracheae (using *DSRF > InR-RNAi*). Reduced tracheation of all tissues did not affect larval development (Figure 6A) or carbohydrate metabolism (Figures S7A–S7C) but resulted in leaner larvae (Figure 6C) with reduced lipid stores (Figure 6D) and increased hemolymph glycerol (a metabolite derived from the hydrolysis of triglycerides) (Figure 6E), consistent with reduced lipid storage capacity in the fat body. These larvae did manage to eclose as adults but were sick and short-lived even in the presence of nutritious food (Figure 6B and data not shown). By contrast, when reduced tracheation was confined to the gut, no developmental or metabolic phenotypes were apparent in larvae (Figures S7D–S7H), and there was no difference in adult lifespan between the experimental flies and controls on nutritious food (Figure 6F). We then hypothesized that the specific effect of nutrient restriction on gut tracheae may fulfil an adaptive role to allow flies to deal with poor nutritional conditions. To test this idea, we exposed the *DSRF>InR-RNAi* flies with reduced gut tracheation to a low-calorie diet throughout their adult lifetime, and found them to be significantly more resistant to nutrient scarcity than control flies: a tracheal phenotype that was confirmed using the recently published tracheal driver *14D03-GAL4* (Guo et al., 2013) (Figure 6G and data not shown). Metabolic profiling of these adult flies revealed no differences in carbohydrate metabolism but showed a reduction in lipid stores in poor nutritional conditions (Figures 6H, 6I, and S7I–S7L).

In summary, manipulations that recapitulate the effects of nutrient restriction and reduced insulin signaling specifically in tracheal terminal cells show that these cells are important metabolic mediators.

## Discussion

### Nutrient-Responsive Neurons as Effectors of Adaptive Tracheal Changes

Our work has uncovered a new mechanism coupling nutrition and metabolism. In response to specific nutritional cues, small subsets of neurons are activated to regulate tracheal branching in an organ-specific and metabolically significant fashion. At least one of the two yeast-responsive neuronal subsets also responds to reduced oxygen—the other environmental modulator of tracheal branching in flies—so it will be interesting to determine the contribution of these neurons to the previously reported tracheal adaptations to hypoxia. Importantly, our identification of a shared neuronal substrate for both nutritional and hypoxic stimuli is, to our knowledge, the first of its kind in invertebrates and one remarkably similar to the mammalian carotid body: a cluster of chemoreceptors that monitors arterial oxygen concentration and nutrient levels to regulate breathing and cardiovascular tone (Pardal and López-Barneo, 2002, Prabhakar, 2000). Future work will aim to establish whether these *Drosophila* neurons are able to sense oxygen and/or nutrients directly and whether they do so using mechanisms akin to those described in the carotid body. This would lend further support to the existence of an evolutionarily conserved link between oxygen and nutrient neuronal sensing.

**Figure 7:**
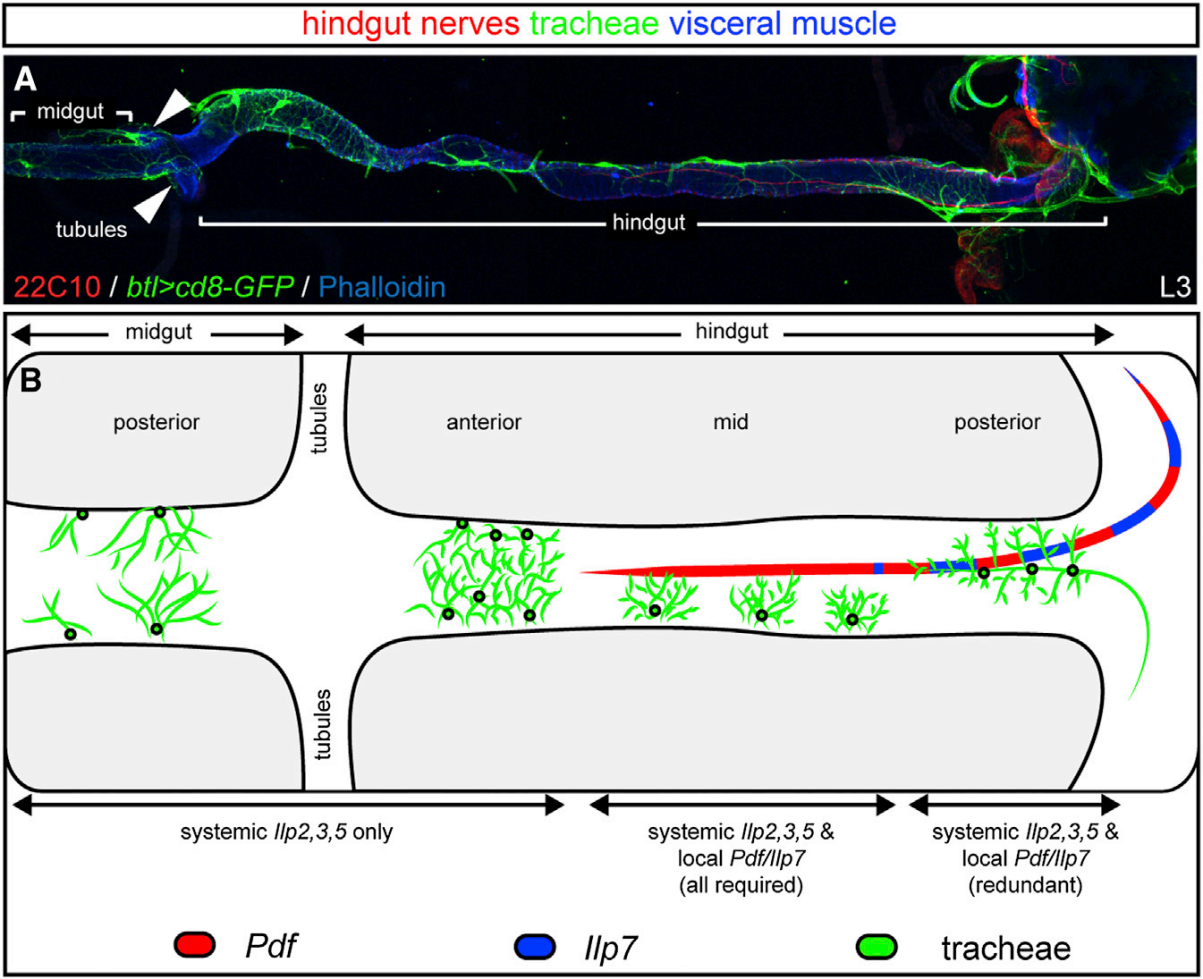
Regional Specificity of the Gut Neuron/Tracheae Interactions (A) The different visceral tracheal terminal branches of the posterior midgut and hindgut, as visualized in green in a 3^rd^-instar larva using a pan-tracheal reporter to express a membrane-tagged GFP (*btl>cd8-GFP*). 22C10 staining (in red) highlights the two hindgut nerves and phalloidin (in blue) labels visceral muscles. (B) Illustration summarizing the different kinds of visceral tracheal terminal cells, their positioning relative to the hindgut nerves, and their regulation by systemic and paracrine neuropeptides at the 3^rd^-instar stage. In the posterior midgut and anterior hindgut, there is no apparent dorsoventral patterning with regard to the positioning of tracheal terminal cells. In these intestinal portions, tracheal terminal growth is exclusively under the control of the systemic mNSC-derived Ilps. In the mid-hindgut, the visceral tracheal terminal cells reach the hindgut from its ventral side and extend branches that eventually cover the dorsal domain. Mutation of *Pdf* or *Ilp7* alone, as well as the triple *Ilp2,3,5* mutation, all lead to reduced branching. In the posterior hindgut, where the Ilp7/Pdf axons abut the posterior hindgut tracheal branches, Ilp7 is partially redundant with Pdf and the systemic Ilps.

Molecularly, the neuronal control of different tracheal subsets involves both local and systemic actions of insulin- and VIP-like neuropeptides: neuronal mechanisms that are particularly complex and combinatorial along the digestive tract (Figure 7) and that differ from the known adaptive target-derived signals that sculpt tissue-specific angiogenesis (Cao, 2007, Fraisl et al., 2009). In this regard, tracheal cells can be seen as “metabolic motor neurons”; as the nervous system modulates motor neuron activity to regulate muscle contraction, it also modulates the branching of tracheal terminal cells to control the metabolic state of cells such as those of the fat body or the gut epithelium. It will be of interest to investigate whether similar mechanisms are deployed in vertebrates to effect long-lasting, tissue-specific and metabolically significant changes in angiogenesis in response to nutrition, in a manner distinct from (but reminiscent of) the acute changes in blood supply effected by neurons by acting on blood vessel musculature (see, for example, Matheson et al., 2000).

### Organ-Specific Regulation of the Tracheal System by Local and Systemic Insulin- and VIP-like Neuropeptides

In *Drosophila*, previous gain- and loss-of-function experiments had failed to reveal unique functions for most of the eight known Ilps (Brogiolo et al., 2001, Grönke et al., 2010). The regional regulation of tracheal subsets hence provides one possible explanation for the apparent redundancy of the *Ilp* gene family in *Drosophila*: while all these Ilps may indeed have the same function (in this case, to modulate tracheal growth in response to nutrition), they may carry it out in different places—for example, in the posterior hindgut in the case of Ilp7 and in other parts of the digestive tract for Ilp2, Ilp3, and Ilp5. This regional control of tracheal growth may extend to other regions: gut visceral musculature and CNS glia are known to activate *Ilp3* and *Ilp2/Ilp6* gene expression respectively in a nutrient-dependent fashion (Chell and Brand, 2010, O’Brien et al., 2011, Sousa-Nunes et al., 2011). In light of our findings and the recent discovery that intestinal tracheae can regulate stem cell proliferation (Li et al., 2013), it is possible that local regulation of tracheal branching by Ilps contributes to their reported action on intestinal or neuronal stem cell proliferation (Chell and Brand, 2010, O’Brien et al., 2011, Sousa-Nunes et al., 2011).

Effects of insulin and VIP on blood vessels have been described in vertebrates (Chaudhuri et al., 2012, Holzer, 2006). Indeed, although the effect of Pdf on intestinal tracheal branching is unexpected in *Drosophila* (where this peptide is known for its central role in clock neurons; Taghert and Nitabach, 2012), neurally derived VIP has a vasodilatory effect on the arterioles of small intestine and colon (Holzer, 2006). However, the physiological significance of these (largely ex vivo) observations has not been entirely elucidated (Matheson et al., 2000). In contrast to this mode of regulation, involving acute modulation of endothelial muscle tone, the evidence for longer-lasting effects of these peptides on angiogenesis—which would be more akin to their action on the *Drosophila* tracheal system—is more tenuous and often contradictory (see, for example, Ogasawara et al., 1999, Ribatti et al., 2007). Our findings suggest that their effects may have been underestimated because they act in partially redundant fashion and in response to specific nutritional cues. Mechanistically, it has been proposed that the vertebrate peptides regulate proangiogenic target-derived signals. By contrast, our tracheae-specific receptor downregulation experiments clearly indicate that these peptides can act directly on the tracheal cells, so it will be of interest to establish whether both modes of action contribute to their effects on vertebrate angiogenesis.

### Metabolic Significance of the Tracheal Nutritional Plasticity

In *Drosophila*, whole-organism manipulations of insulin signaling such as ablation of insulin-producing cells or Ilp mutation result in both slower development and “diabetic” phenotypes, highlighting their dual insulin/IGF-like role (Grönke et al., 2010, Rulifson et al., 2002). Strikingly, downregulation of insulin signaling only in one cellular target—the tracheal terminal cells—uncouples the developmental from the metabolic phenotypes of these peptides, thus identifying the tracheal system as an important and previously unrecognized metabolic target of insulin signaling in the fly. Hence, the tracheal involvement in previously reported insulin-modulated phenotypes, such as lifespan or resistance to oxidative stress (Grönke et al., 2010), deserves further investigation. Interestingly, a pan-tracheal reduction in insulin signaling results in normal carbohydrate metabolism but leads to reduced adiposity. This is suggestive of abnormal lipid metabolism in the fat body and is consistent with the recent finding that reduced fat tissue vascularity leads to fat mass reduction without affecting glucose homeostasis in young mice (Sung et al., 2013)—although in both mice and flies this phenotype may eventually prove to be deleterious (Sung et al., 2013 and Figure 6E). Reduced adiposity is a phenotype that, although also consistent with one of the classic symptoms of type I diabetes in humans, had not previously been observed in flies with a ubiquitous reduction in insulin signaling or lacking the systemic Ilp peptides (puzzlingly, these flies were actually found to accumulate triglyceride; Böhni et al., 1999, Grönke et al., 2010). We suggest that this increased adiposity may have been secondary to the IGF-like effects of Ilps on developmental time, and only by uncoupling these developmental from the metabolic effects of Ilps, as we have done with the tracheal-specific reduction of insulin signaling, can some of the “true insulin-like” phenotypes of Ilps be unmasked.

We have also found that subtle changes in insulin signaling or in the nutritional content of the fly’s diet (some of which are within the range of those normally found in diets used for fly rearing in different labs) have a striking effect on an unexpected tracheal population: that of the digestive tract. It will be of interest to explore the cellular mechanisms underlying their differential sensitivity. These might result from differences in receptor levels or composition—the Ret-like receptor tyrosine kinase Stitcher, recently shown to synergize with InR in mitotic tissues, is a possible candidate (O’Farrell et al., 2013). Alternatively, it could be caused by differences in downstream signaling components such as Foxo, which has been shown to account for some organ-specific responses (Tang et al., 2011).

Functionally, by uncovering gut-specific effects of tracheation on adult survival and lipid mobilization upon nutrient scarcity, we have identified the tracheal system as a possible anatomical substrate for the previously reported effects of nutrient acquisition during developmental and growth periods on a variety of adult features (Foley and Luckinbill, 2001, Zwaan et al., 1991). Enterocytes would appear to be the obvious cellular mediators of these effects; changes in oxygen supply may modulate the metabolic state of these absorptive cells, and long-term adaptations to nutrient scarcity may result from differential nutrient absorption and/or utilization. However, enterocytes need not be the only intestinal targets of the nutrient-driven tracheal changes: the tracheal regulation of stem cell proliferation described above (Li et al., 2013) provides an alternative (or additional) target. Consistent with this idea, there is correlative as well as (more limited) functional data implicating neuronal factors in the regulation of angiogenesis in tumor environments (Jang et al., 2000, Madden et al., 2011, Toda et al., 2008). Furthermore, oxygen need not be the sole mediator of the gut tracheae-driven adaptations: Li et al. (2013) also found that tracheae produce Dpp, an important TGFβ-like signaling molecule. In future, it will be of interest to explore not only these intestinal targets, but also whether the intestinal tracheal plasticity is more widely regulated by other environmental stimuli—such as gut epithelial infection or damage. From a more translational perspective, most studies of adaptive angiogenesis in vertebrates have focused on the adipose vasculature (Cao, 2010, Lijnen, 2008). In light of our *Drosophila* findings, it will be of interest to explore the nutritional plasticity of the gastrointestinal vasculature, as well as its contribution to pathologies such as obesity or to the metabolic improvements following gastric bypass interventions.

## Experimental Procedures

### Visualization and Scoring of Tracheal Growth

Tracheae were imaged and blindly scored using DIC optics (see Extended Experimental Procedures for details). Quantifications were performed as follows:

#### Body Wall Tracheae

The stereotypical endings of the third dorsal branch, directly posterior to the large tracheal commissure on the third segment, were counted as described in (Centanin et al., 2008).

#### CNS Tracheae

Tracheal coverage was quantified in the VNC—a relatively flat tissue with well-defined anatomical boundaries—as the ratio between the total length of tracheal arbours (which are complex and nonstereotypical) divided by total VNC area (μm/μm^2^). Tracheal length was measured using a custom-written ImageJ macro (Schneider et al., 2012). After median filtering (radius = 3 pixels) to reduce image noise, a polygonal region of interest (ROI) was manually drawn to mark the tissue area. Following background subtraction to enhance the visibility of tracheae, the image was segmented and the tracheal area within the ROI was measured.

#### Gut Tracheae

In the mid-hindgut, where the tissue surface and three-dimensional properties allowed semiautomated quantification, the same procedure as for the VNC was used, but the segmented image was subsequently skeletonized. Parts of gut tissue wrongly identified as tracheae or segments of the tracheal tree missed by the program were manually edited before counting the total number of pixels in the skeletonized tracheal tree. In other intestinal portions, where the ruggedness and/or bends and twists of the target tissue made semiautomated quantification impractical, tracheal coverage was blindly scored using Likert-type scales ranging from no difference to wild-type (3) to strongly increased (5) or strongly reduced (1) (see Figure 1 legend for color coding of displays). The validity of this scoring system was confirmed in the body wall and mid-hindgut, where Likert-quantified scores were comparable to those obtained by counting or by semiautomated quantification respectively (data not shown). Likert rank data were displayed as the mean (circled) on diverging stacked bar charts, with the percentage of samples assigned to each Likert rank reflected in the length of each differently colored segment.

We refer to Extended Experimental Procedures for details of statistical analyses, fly stocks, diets, and more standard methods (immunohistochemistry, transmission electron microscopy, metabolic assays, survival assays, developmental rate and size quantifications, and in vivo recordings of neuronal activity).

Extended Experimental Procedures **Fly Stocks and Rearing**

The following fly stocks were used: *DSRF-GAL4* (Gervais and Casanova, 2011), *UAS-PI3K-DN* (*UAS-Dp110*^*D954A*^; Leevers et al., 1996), *UAS-InR-RNAi* (VDRC stock 992), *UAS-btl-RNAi* (VDRC stock 110277), *Ilp7*^*211*^*, Ilp2,3,5* and *Ilp2,3,5*,7 mutants (Grönke et al., 2010), *btl-GAL4* (Shiga et al., 1996), *UAS-cd8-GFP* (Lee and Luo, 1999), *Ilp7-GAL4* (Yang et al., 2008), *UAS-kir2.1* (Baines et al., 2001), *Pdf*^*01*^ mutants (Renn et al., 1999), *UAS-Pdfr-RNAi* (TRiP stock HMS01815), *Va-GAL4* (Suska et al., 2011), *UAS-GCaMP3* (Tian et al., 2009), *UAS-myr-mRFP* (hop B2, following re-mobilization of the original hop contributed to the Bloomington Stock Center by H. Chang), *Va-GAL4* (Allan et al., 2003) and *UAS-TrpA1* (Hamada et al., 2008). *Oregon R* (*OreR*) were used as wild-type flies. In genetic experiments involving the *GAL4-UAS* system, two parental controls were used: the *GAL4* driver and the *UAS* drivers separately crossed to *w*^*1118*^ flies. An isogenic w^*1118*^ stock was used as control for the experiments involving the *Pdf*^*01*^ mutant, and all *Ilp* mutants were backcrossed for at least 8 generations to a large population of *w*^*Dah*^ flies, which was used as a control strain in all the *Ilp* mutant experiments. All crosses and experiments were kept at 25°C, including those designed to achieve persistent, low-level activation of the Ilp7 neurons by expression of TrpA1.

To obtain larvae of the desired developmental stage, a small number of flies (less than 10 virgin females) were housed in embryo collection cages with apple juice plates topped with abundant yeast (see section below). Following frequent plate changes, a 4 hr egg lay was collected. Eggs/larvae were allowed to develop until the desired stage depending on the experiment, but they were typically screened at 72h after egg laying (AEL) to control for developmental delay and discard larvae that had not reached 3^rd^-instar stage. At this point, larvae were transferred to fresh plates in controlled numbers (25 per plate) to avoid nutrient restriction resulting from overcrowding.

For the experiments requiring adult flies, an equal volume of eggs (29 μl, corresponding to ca. 200 eggs) was squirted into bottles to control for the density of developing larvae and synchronize the emergence of adults. Adult flies were maintained in vials at low densities (10–15 flies per vial) and were transferred to fresh food vials every 2–3 days.

**Diets**

Our stocks are normally reared on a standard cornmeal/agar diet (5.5% cornmeal, 6% dextrose, 1.3% yeast, 0. 55% agar supplemented with 0.18% Nipagin and 2.9 ml/l propionic acid). For most larval experiments, larvae were raised and staged under nutrient-rich conditions using apple juice plates (2.3% agar, 2.3% dextrose, 25% apple juice in dH_2_O) with abundant baker’s yeast (prepared fresh every time using 5 g of yeast and 10 ml dH_2_O). For the experiments assessing the effect of yeast concentration on larval tracheal growth (Figure 1), the standard cornmeal/agar diet above was used with the following three yeast concentrations: 8% (nutritious), 2% (mild nutrient restriction) or 0.8% (severe nutrient restriction). These same diets were used to assess the effect of larval diet on adult tracheation; “control” flies (Figure 1J) were raised and maintained in the nutritious cornmeal/agar diet with 8% yeast, whereas flies that were nutrient restricted as larvae (Figure 1K) were raised in the cornmeal/agar diet with 0.8% yeast, and then were switched to the nutritious cornmeal/agar diet with 8% yeast after eclosion.

For the adult survival or metabolic experiments, flies of the different genotypes were reared and aged for 1 day in standard food, and were then transferred to a more defined diet with the same sugar/yeast ratio (60%/40%) but differing nutritional content: nutritious (5.4% sucrose and 3.6% yeast in 1% agar, 0.18% Nipagin), or restricted (0.9% sucrose and 0.6% yeast in 1% agar, 0.18% Nipagin). Metabolic assays were conducted as described below 7 days after feeding on either diet.

**Visualization and Scoring of Tracheal Growth**

For visualization of tracheae using DIC optics, larval or adult tissues were dissected in 100% glycerol and were immediately mounted and used to quantify/image phenotypes using a Zeiss Axioplan equipped with a Leica DFC425C camera. Tracheal coverage was measured in specific regions of the gut, brain or body wall. The criterion for defining larval gut regions was typically their proximity to an obvious landmark: e.g., the gastric caeca (anterior midgut) or the Malpighian tubules (posterior midgut). Gut and brain tracheae were scored in early 3^rd^-instar larvae (72 hr AEL), whereas body wall tracheae were scored in mid-3^rd^–instar larvae as previously done by (Centanin et al., 2008). In the cases where it was necessary to correct for developmental delay (*Ilp2,3,5* mutants and severe nutrient restriction), the developmental delay was first monitored and found to be ca. 24 hr at 72 hr AEL. Egg collections and transfers to fresh plates were thus carried out 24 hr earlier, so as to obtain 3^rd^-instar larvae at the same time as controls. When comparing genotypes, mutants and controls were mounted and processed on the same slides. In CNS images, anterior is to the top. In body wall images, anterior is to the top and the right dorsal terminal branch is always shown. In all gut images, anterior (oral) is to the left.

Tracheal scoring was performed as described in the Experimental Procedures. Tracheal coverage data were tested for normality using the Shapiro-Wilk test. Because most distributions were found to be nonnormal, coverage data were analyzed using nonparametric statistics. The Wilcoxon rank-sum test was used for pairwise comparisons, and was preceded by a Kruskal-Wallis test if two parental controls were analyzed. Multiple comparisons were performed with the Kruskal-Wallis test and the Wilcoxon test with the Benjamini and Hochberg/Yekutieli procedure for controlling false discovery rate. In all figures, significance levels are indicated with asterisks as follows: p < 0.05 ^∗^, p < 0.01 ^∗∗^, p < 0.001 ^∗∗∗^.

**Developmental Rate and Size Quantifications**

To assess developmental rate, 4 hr lays were collected and aged as described in the fly rearing section. By 72 hr AEL, the percentage of larvae reaching the 3^rd^-instar stage (as assessed by the anatomy of their mouth hooks and anterior spiracles) was comparable across genotypes. These 3^rd^-instar larvae were transferred to fresh apple juice plates with yeast in groups of 10, and the emergence of pupae was scored hourly. Statistical analysis was performed using survival statistics (Harrington and Fleming, 1982).

The size of mid-3^rd^-instar larvae (96 hr AEL) larvae was determined following their incubation in a 60°C water bath for 20 min, which relaxed all muscles. The dead larvae were immediately imaged in groups of three with a Leica MZ16F equipped with a DFC420C camera. Size measurements were done semi-automatically using ImageJ.

**Survival Assays**

Upon eclosion, experimental flies reared in noncrowding conditions were collected daily and allowed to mate for a further 24 hr to avoid multiple matings. The flies were then separated into males and females and were kept as described in the fly rearing section (see Extended Experimental Procedures). Tubes were scored daily for dead flies. Male and female survival data were analyzed separately as described in (Harrington and Fleming, 1982). Since no differences between males and females were found, only the data for male flies is shown in Figure 3.

**Metabolic Assays**

For whole-animal measurements, metabolic assays were performed on fresh (not previously frozen) 96 hr AEL larvae or adult males (previously frozen in liquid nitrogen). Groups of 10 adult males or larvae (previously washed in dH_2_0, then briefly dried on tissue paper) were put in a 2 ml Eppendorf tube containing a steel bead and 500 μl (or 200 μl for adult TAG assays) grinding buffer (PBS for carbohydrate assays and PBS with 0.05% Tween for the TAG assays). Grinding was performed twice for 1 min at 30Hz using a QIAGEN TissueLyserII, after which the samples were immediately incubated at 70°C for 5 min. Samples were then transferred to ice. For the carbohydrate assays, the samples were centrifuged for 3 min at 13,000 g. For each sample, 25 μl of supernatant was added to 25 μl PBS, PBS with amyloglucosidase or PBS with trehalase for carbohydrate digestion in 96-well plates. Samples were incubated for 2 hr at 37°C degrees. 10 μl were then transferred to 100 μl of glucose assay reagent (Sigma) or Protein Quantification Kit – Rapid (Sigma). For TAG assays, 25 μl of each sample were added to 25 μl PBS or Triglyceride reagent (Sigma). Following a 30 min incubation at 37°C, samples were centrifuged for 3 min at 13,000 g, and 10 μl of each sample were transferred to individual wells in 96-well plates containing 100 μl of free glycerol reagent (Sigma) or Protein Quantification Kit – Rapid (Sigma). Absorbance was read according to manufacturer’s description using a Perkin Elmer Wallac Victor 1420 and a Bio-Rad Model 680 Microplate Reader. Concentration values were calculated using standard curves, and the protein values were used to normalize the TAG and sugar assays.

Hemolymph measurements were performed in a similar fashion. Groups of 10 cleaned and dried larvae were torn open with forceps on a clean glass slide. To measure trehalose, 2 μl of hemolymph were diluted in 38 μl of PBS and the mix was immediately heated to 70°C. Following a 10 min centrifugation step at 13,000 *g*, 10 μl were added to 30 μl of PBS or PBS with trehalase. The remaining steps were performed as described above for the whole-animal assays without the protein normalization. To measure hemolymph free glycerol, hemolymph was extracted as described for trehalose and 2 μl were diluted in 10 μl PBS. Samples were then heated and centrifugated in an identical fashion, and 10 μl of each sample were added to single wells in a 96-well plate containing 100 μl of free Glycerol Reagent (Sigma).

All metabolic data were tested for normality using the Shapiro-Wilk test. Because most distributions were found to be nonnormal, data were analyzed using nonparametric statistics. The Wilcoxon rank-sum test was used for pairwise comparisons, and was preceded by a Kruskal-Wallis test if two parental controls were analyzed. In all figures, significance levels are indicated with asterisks as follows: p < 0.05 ^∗^, p < 0.01 ^∗∗^, p < 0.001 ^∗∗∗^.

**Immunohistochemistry**

Larval guts were dissected in PBS and fixed in 4% paraformaldehyde for 20 min. Subsequent washes and incubations were done in PBS with 0.2% Triton. Tissues were incubated overnight with primary antibody at 4°C, followed by a 2 hr incubation with secondary antibodies at room temperature the next day. The following antibodies were used: mouse α-futsch (22C10, Developmental Studies Hybridoma Bank; 1:10), goat α-green fluorescent protein (GFP) (Abcam, 1:2,000), rabbit α-Ilp7 (Miguel-Aliaga et al., 2008; 1:5,000), mouse anti-Pdf propeptide (Pdf C7, Developmental Studies Hybridoma Bank; 1:50) and mouse anti-DSRF antibody (Active Motif, 1:10). Phalloidin-Cy5 (Molecular Probes) was used at 1:200. FITC-, Cy3-, and Cy5-conjugated secondary antibodies were obtained from Jackson Immunolabs and used at 1:200 (1:100 for the Cy5-conjugated antibody). Fluorescent preparations were mounted in Vectashield with DAPI (Vector Labs) to visualize all nuclei. Images were acquired using a Leica SP5 confocal microscope.

**Transmission Electron Microscopy**

Transmission electron microscopy was performed using standard techniques. Briefly, guts were dissected and fixed overnight at 4°C in 2.5% glutaraldehyde in 0.1 M phosphate buffer. The next day the guts were postfixed for 30 min in 1% osmium tetroxide in 2% glutaraldehyde in 0.1 M phosphate buffer, and for a further 1 hr in 2% osmium tetroxide. Guts were then bulk-stained in 2% uranylacetate and embedded in araldite after dehydration steps. Ultrathin sections were cut using a Leica UC6 ultramicrotome (Leica Microsystems) mounted on 100-mesh copper grids. These sections were double-stained for 10 min with 2% uranylacetate in 70% methanol, followed by 5 min lead citrate in water. Imaging was performed using a FEI-Tecnai 12 Biotwin (100 kV, tungsten filament) and a F214A digital camera.

**In Vivo Recordings of Neuronal Activity**

Immobilized 72 hr 3rd-instar larvae expressing UAS-GCaMP3, UAS-myr-mRFP from either *Ilp7-GAL4* or *Va-GAL4* reared on apple juice plates with yeast were used for these experiments. Ca^2+^ imaging of nutrient and oxygen responses was performed essentially as described for *C. elegans* worms (Busch et al., 2012). Briefly, larvae were immobilized on agar pads (2% in H_2_O) with Dermabond tissue adhesive (Ethicon), and were individually housed by placing a 6x6 mm wide and 0.2 mm deep polydimethylsiloxane microfluidics chamber over them. For the hypoxia experiments, humidified gas mixtures (BOC) of 21% or 1% O2 (balance N2) were continuously delivered to the microfluidics device at 2.5 ml min-1 from a syringe pump. Teflon valves (Automate Scientific) were used to rapidly switch between the two gas streams every 120 s, as described in (Busch et al., 2012). For the nutrition experiments, circa 2 min after the start of each recording, larvae were presented with a suspension of liquid yeast (1 g:3 ml in dH2O) or 9% sucrose in dH_2_0, pumped in through PE50 polyethylene tubing (BD intramedic) from a syringe. The time when the yeast reached the chamber was manually recorded and was used to realign traces to compare and average the responses of different larvae. Ilp7 or Va neurons were recorded with a 100 ms exposure time in 0.5 s intervals on an inverted compound microscope (Axiovert, Zeiss), using a 2.0 neutral density filter, a GFP/DsRed emission filter set (Chroma), a 40x C-Apochromat lens (Zeiss), a Dual-View beamsplitter with GFP/DsRed emission filters (Photometrics) and a Cascade II 512 EMCCD camera (Photometrics). Images were binned 2x2. The fluorescence intensity of individual neurons was tracked over time using a region of interest in both the GCaMP and RFP channels with custom-written software in MatLab (Mathworks). From each region of interest, the 20 brightest pixels were selected, median pixel intensity was subtracted for each channel separately, and the GCaMP/RFP fluorescence ratio was then determined to normalize the GCaMP signal for Z axis drift. Mean fluorescence ratio plots and traces of individual responses were generated in Excel.

## Supplemental Information

Movie S1. Representative Movie Illustrating the Ilp7 Neuron Response to Yeast, Only GCaMP Channel Is Shown, Related to Figure 5

Movie S2. Representative Movie Illustrating the Ilp7 Neuron Response to Reduced O_2_ Availability, Only GCaMP Channel Is Shown, Related to Figure 5

Document S1. Article plus Supplemental Information
